# Notch signaling pathway in cancer: from mechanistic insights to targeted therapies

**DOI:** 10.1038/s41392-024-01828-x

**Published:** 2024-05-27

**Authors:** Qingmiao Shi, Chen Xue, Yifan Zeng, Xin Yuan, Qingfei Chu, Shuwen Jiang, Jinzhi Wang, Yaqi Zhang, Danhua Zhu, Lanjuan Li

**Affiliations:** State Key Laboratory for Diagnosis and Treatment of Infectious Diseases, National Clinical Research Center for Infectious Diseases, National Medical Center for Infectious Diseases, Collaborative Innovation Center for Diagnosis and Treatment of Infectious Diseases, The First Affiliated Hospital, Zhejiang University School of Medicine, Hangzhou, 310003 China

**Keywords:** Cancer therapy, Tumour biomarkers

## Abstract

Notch signaling, renowned for its role in regulating cell fate, organ development, and tissue homeostasis across metazoans, is highly conserved throughout evolution. The Notch receptor and its ligands are transmembrane proteins containing epidermal growth factor-like repeat sequences, typically necessitating receptor-ligand interaction to initiate classical Notch signaling transduction. Accumulating evidence indicates that the Notch signaling pathway serves as both an oncogenic factor and a tumor suppressor in various cancer types. Dysregulation of this pathway promotes epithelial-mesenchymal transition and angiogenesis in malignancies, closely linked to cancer proliferation, invasion, and metastasis. Furthermore, the Notch signaling pathway contributes to maintaining stem-like properties in cancer cells, thereby enhancing cancer invasiveness. The regulatory role of the Notch signaling pathway in cancer metabolic reprogramming and the tumor microenvironment suggests its pivotal involvement in balancing oncogenic and tumor suppressive effects. Moreover, the Notch signaling pathway is implicated in conferring chemoresistance to tumor cells. Therefore, a comprehensive understanding of these biological processes is crucial for developing innovative therapeutic strategies targeting Notch signaling. This review focuses on the research progress of the Notch signaling pathway in cancers, providing in-depth insights into the potential mechanisms of Notch signaling regulation in the occurrence and progression of cancer. Additionally, the review summarizes pharmaceutical clinical trials targeting Notch signaling for cancer therapy, aiming to offer new insights into therapeutic strategies for human malignancies.

## Introduction

The Notch locus was initially identified in 1917 through genetic studies involving a mutant strain of *Drosophila melanogaster* exhibiting notched wings.^1^ The Drosophila Notch gene was subsequently isolated in 1983.^2^ It was later revealed that the protein encoded by the *Notch* gene functions as a transmembrane receptor with multiple epidermal growth factor (EGF)-like repeats, typically activated by transmembrane ligands expressed on adjacent cells.^3^ To date, Notch receptors and ligands have been discovered in various metazoans, serving as integral components of the Notch signaling cascade and participating in diverse biological processes such as cell fate determination, embryonic development, organ formation, and tissue repair.^4,5^

Extensive research has been conducted on Notch signaling pathway, investigating its role as an oncogene or tumor suppressor in various cellular contexts. The involvement of the Notch signaling pathway in human malignancies was initially elucidated in T cell acute lymphoblastic leukemia (T-ALL), where the chromosomal translocation t(7;9) (q34;q34.3) results in the truncation of Notch1 transcripts.^6^ Subsequent cancer genome sequencing has unveiled widespread oncogenic Notch gene mutations in diverse human cancers, such as cutaneous and lung squamous cell carcinoma (LUSC),^7^ breast cancer,^8^ anaplastic large cell lymphoma,^9^ and chronic lymphocytic leukemia (CLL).^10^ Moreover, accumulating evidence indicates that the dysregulation of the Notch signaling pathway intricately controls the onset and progression of hematologic malignancies and solid tumors in humans.^11,12^ This occurs through complex mechanisms, including tumor angiogenesis, modulation of the immune microenvironment, epithelial-mesenchymal transition (EMT), cancer energy metabolism, and resistance to chemotherapy. For instance, oncogenic Notch signaling facilitates T-ALL cell proliferation by activating nuclear factor-kappa B (NF-κB) through Asb2 mediation.^13^ Additionally, activated Notch signaling contributes to the acquisition of stem-like properties in esophageal adenocarcinoma.^14^ The pivotal role of Notch signaling in cancer biology has garnered significant attention, leading to the exploration of targeted cancer therapies based on Notch signaling. This review offers a systematic exploration of the research advancements in the Notch signaling pathway within the context of tumors. It concentrates on unraveling the molecular mechanisms underlying Notch signaling-mediated tumorigenesis and progression. Furthermore, the review outlines targeted therapeutic strategies for tumors that are rooted in Notch signaling, as evidenced by clinical research endeavors. The systematic insights provided in this review aim to furnish a current and thorough understanding of the Notch signaling pathway in tumors. This knowledge is expected to contribute significantly to the future development of the Notch signaling pathway in both basic research and clinical translation.

## Overview of the Notch signaling pathway

**Fig. 1:**
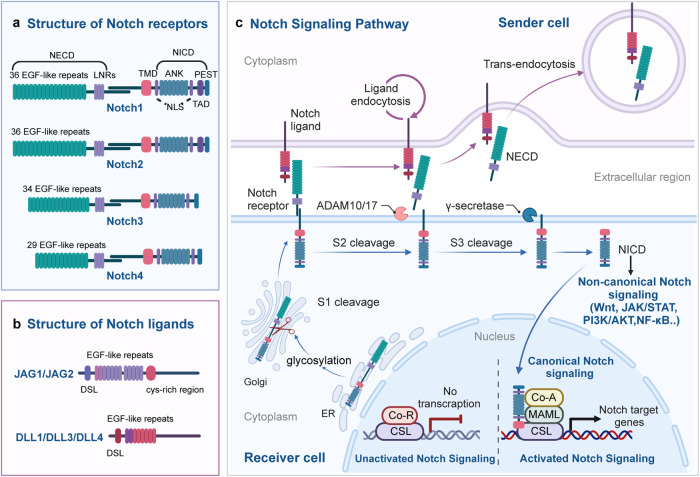
Notch signaling overview. **a** Four Notch receptors (Notch1, Notch2, Notch3, and Notch4) and their respective structures. **b** Structures of five Notch ligands (JAG1, JAG2, DLL1, DLL3, and DLL4). **c** Schematic representation of canonical and non-canonical Notch signaling pathways. (Figure created using BioRender.com). NECD Notch extracellular domain, EGF epidermal growth factor, LNRs Lin12-Notch repeats, TMD transmembrane domain, NICD Notch intracellular domain, ANK ankyrin repeat, NLS nuclear localization sequences, TAD transcription activation domain, PEST proline/glutamic acid/serine/threonine, CSL CBF1/suppressor of hairless/Lag1, ADAM a disintegrin and metalloprotease, ER endoplasmic reticulum, Co-R corepressor, CSL CBF1/suppressor of hairless/Lag1, Co-A coactivator, MAML mastermind-like

The Notch signaling pathway exhibits high conservation throughout evolution, coordinating multiple physiological mechanisms during development and homeostasis in metazoans. Classically, ligand-activated Notch receptors initiate transcription of downstream target genes by interacting with the DNA-bound CSL-co-repressor complex, forming the canonical Notch signaling pathway. Over the years, mounting evidence has demonstrated that Notch can function via non-canonical pathway that is independent of ligands or CSL.^15,16^ The canonical Notch signaling pathway plays a major physiological function in intercellular interaction and gene transcription regulation, while non-canonical Notch signaling involves the crosstalk between various signaling pathways to execute activation of target genes.^17^ In this overview, we provide a brief summary of the key components of the Notch signaling pathway and examine the mechanisms underlying both the canonical and non-canonical Notch signaling pathways (Fig. 1).

### Components of the Notch signaling pathway

The mammalian Notch signaling pathway comprises three principal components: Notch receptors, ligands that bind to Notch receptors, and downstream effectors of Notch signaling.^18^ In *D. melanogaster*, there is a single Notch receptor ortholog, Notch1.^19^ However, in mammals, three additional Notch receptors exist: Notch2, Notch3, and Notch4. The Notch receptor is a transmembrane protein with three main segments: the Notch extracellular domain (NECD), transmembrane domain (TMD), and Notch intracellular domain (NICD).^20^ The NECD contains multiple EGF-like repeats and a negative regulatory region (NRR), modified by O-glycans to regulate the Notch receptor’s affinity for different ligands.^21^ Notch1–4 NECDs have 36, 36, 34, and 29 EGF-like repeats, respectively, crucial for ligand interaction.^22^ The NRR comprises three cysteine-rich Lin12-Notch repeats, stabilizing NECD and membrane-bound NICD interaction, essential for receptor cleavage.^23–25^ The TMD includes an extracellular short region and conserved cysteine residues forming heterodimers.^26^ The NICD consists of an RBPJ [recombination signal binding protein-J] association module (RAM) domain, seven ankyrin repeat (ANK) domains, and two nuclear localization sequences (NLS) on each side of the ANK domain.^27^ Notch1 and Notch2 have a transcription activation domain (TAD) after the ANK sequence, while Notch3 and Notch4 lack a TAD. The C-terminal of NICD has a “PEST” sequence, rich in proline, glutamic acid, serine, and threonine, crucial for NICD stability.^28^

Humans and mice possess five ligands binding to extracellular Notch receptor fragments. Based on the presence or absence of the cysteine-rich region, Notch ligands are categorized into Serrate-like ligands Jagged1 (JAG1) and JAG2, and delta-like ligands DLL1, DLL3, and DLL4.^29^ Notch ligands are cell membrane proteins, sharing structural similarities with the Notch receptor. The extracellular domains of JAG1/2 consist of the DSL [i.e., Delta, Serrate, and LAG-2] domain, EGF-like repeats, and a cysteine-rich region.^30^ The extracellular domains of DLL1/3/4 are akin to JAG1/2 but lack the cysteine-rich region.

### The canonical Notch signaling pathway

The canonical Notch signaling pathway involves a series of intricate steps in the maturation and activation of Notch proteins. Initially, Notch proteins are transported to the endoplasmic reticulum as single-stranded precursors. Within the endoplasmic reticulum, the EGF-like domain of the Notch receptor undergoes glycosylation.^31,32^ The glycosylated Notch single-chain precursor is then transported to the Golgi apparatus. In the Golgi apparatus, a furin-like convertase cleaves the S1 site in the extracellular segment of the Notch transmembrane region, resulting in the formation of two distinct fragments: the NECD and the TMD.^33,34^ These fragments subsequently combine through a Ca^2+^-dependent non-covalent bond, forming the mature Notch receptor in the shape of a heterodimer. The mature Notch receptor, now a type I transmembrane protein, is then transported to the cell surface. Upon reaching the cell surface, the Notch heterodimeric transmembrane receptor binds to the Notch transmembrane ligand present on adjacent cells. The S2 cleavage site of the Notch receptor is then cleaved by members of the ADAM (a disintegrin and metalloprotease) metalloproteinase family, specifically ADAM10 or ADAM17.^35,36^ This cleavage releases a partial extracellular fragment, creating a transient intermediate peptide called ‘NeXT’ [Notch extracellular truncation], which consists of the TMD and NICD. The next step involves presenilin-dependent γ-secretase cleaving NeXT at the S3 cleavage site.^37^ This process leads to the release of the soluble NICD of Notch. Subsequently, NICD translocates to the cellular nucleus, where its RAM domain interacts with the transcription factor CBF1/suppressor of hairless/Lag1 (CSL, also called RBPJ).^38^ This interaction facilitates the recruitment of co-activator complexes to CSL, including mammalian mastermind-like 1–3 (MAML1–3) proteins. The assembly of these complexes transforms the original “co-repressor complex” into a “co-activator complex,” resulting in the formation of a multi-protein-DNA complex. This complex promotes the transcription of Notch target genes. In the absence of NICD binding, CSL downregulates the expression of target genes by recruiting various co-repressor proteins.^39^

### The non-canonical Notch signaling pathway

In addition to its interaction with CSL, Notch signaling can influence the expression of related genes through non-CSL-dependent regulatory pathways, constituting the non-canonical Notch signaling pathway.^40,41^ This pathway may be initiated by ligand-independent mechanisms and might not necessitate Notch receptor cleavage. In vertebrates, non-canonical Notch target activation is primarily observed in lineage-restricted progenitors, fate-specific differentiation, and tumorigenesis.^42^ Notably, studies have revealed that Notch can modulate the Wnt/β-catenin signaling pathway,^43^ the Janus kinase/signal transducer and activator of transcription (JAK/STAT) pathway,^44^ the phosphoinositide 3-kinase/protein kinase B (PI3K/AKT) pathway,^45^ and the NF-κB pathway at the post-translational level, thereby exerting its non-canonical biological functions. In human breast epithelial cells, Notch-induced expression of Wnt signaling receptor FZD7 requires non-canonical Notch3 Signaling.^46^ Non-canonical Notch signaling triggered IL-6/JAK/STAT signaling in breast cancer cells and is regulated by IKKα/IKKβ of the NF-κB signaling cascade.^47^ Additionally, recent studies revealed that the non-canonical Notch signaling cascade, mediated by extracellular vesicles and independent of classical ligand-receptor interactions, may have important implications in the invasive phenotype of breast cancer.^48,49^ Lee and colleagues discovered that non-canonical Notch signaling interacted with PTEN-induced kinase 1 (PINK1) to impact mitochondrial function and activate mammalian target of rapamycin complex 2 (mTORC2)/AKT signaling, which maintained brain tumor-forming stem cells.^50^ Perumalsamy et al. identified a novel Notch-mediated non-canonical signaling cascade independent of CBF1/RBPJ, where NICD interacts with the mTOR-Rictor complex, leading to the activation of AKT/PKB to control mammalian cell survival.^51^ During tumorigenesis and progression, the focus on non-canonical Notch signaling activation is growing due to its significance for tumor cellular function, such as proliferation, neoplastic transformation, and inhibition of apoptosis. For instance, non-canonical activation of Notch1 protein sustained the proliferation of melanoma cells, while non-canonical Notch3 signaling could trigger endothelial cell apoptosis to restrict tumor angiogenesis.^52,53^ These non-classical mechanisms allow evolutionarily conserved Notch signaling to carry out more specific functions and may uncover new therapeutic targets as additional mechanisms are revealed in cancers.

## The Notch signaling pathway and cancer

**Fig. 2:**
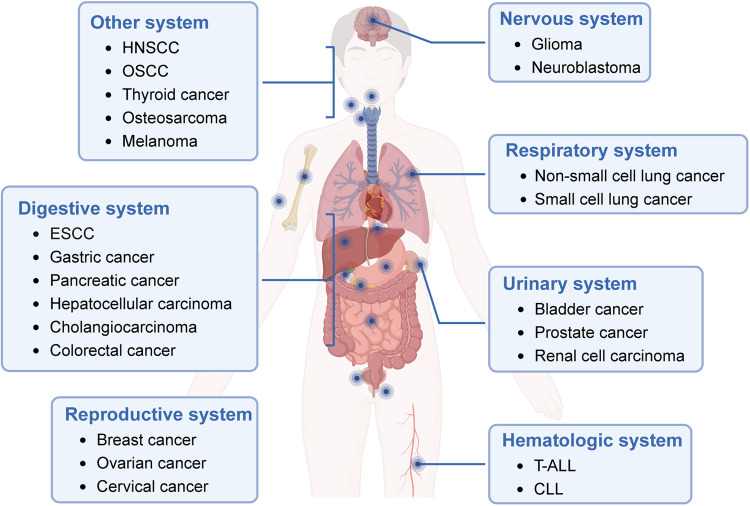
Involvement of Notch signaling in the regulation of diverse cancers. Notch signaling plays a role in the regulation of various cancers, encompassing digestive system tumors, respiratory system tumors, hematological malignancies, urinary system tumors, reproductive system tumors, nervous system neoplasms, and tumors in other systems. (Figure created using BioRender.com)

The Notch signaling pathway plays a crucial role in regulating cell fate decisions under physiological conditions, influencing cell proliferation, differentiation, development, and homeostasis. However, dysregulation of the Notch signaling pathway has been increasingly observed in various human malignancies,^54,55^ spanning digestive system tumors, respiratory system tumors, hematological malignancies, urinary system tumors, reproductive system tumors, nervous system neoplasms, and tumors in other systems (Fig. 2). In this section, we provide a summary of the expression of Notch receptors and ligands in different types of tumors, along with their associations with clinicopathological features and prognosis (Table 1). Furthermore, we review the functions of the dysregulated Notch signaling pathway in different tumors, with the objective of identifying novel diagnostic and prognostic biomarkers based on Notch signaling.

**Table 1:** Expression and clinical features of Notch receptor or ligand in cancers

Category	Cancer type	Notch receptor/ligand	Expression (tumor vs. normal)	Clinical feature	Prognosis	Ref.
Digestive system	CRC	Notch1	Upregulated	Lymph node metastasis, tumor stage, depth of infiltration, histological differentiation	Poor	^58^
	CRC	Notch2	Downregulated	OS	Favorable	^59^
	CRC	Notch3	Upregulated	Tumor differentiation status, tumor recurrence, distant relapse-free survival	Poor	^60^
	CRC	Notch4	Downregulated	Tumor differentiation, invasion, node metastasis, disease-free and OS	Favorable	^61^
	CRC	JAG1	Upregulated	OS, relapse-free survival	Poor	^62^
	CRC	JAG2	Upregulated	Clinical stages	Poor	^63^
	CRC	DLL4	Upregulated	OS, perineural invasion, distant metastasis	Poor	^64^
	HCC	Notch1, Notch4	Upregulated	Disease-specific survival	Poor	^69^
	HCC	Notch2	Upregulated	Clinical stages	Poor	^71^
	HCC	Notch3	Upregulated	Tumor size, TNM stage, OS	Poor	^74^
	HCC	JAG1	Upregulated	Differentiation grade	Poor	^76^
	HCC	JAG2	Upregulated	Intrahepatic metastasis, histological grade, TNM stage	Poor	^78^
	ESCC	Notch1	Upregulated	Tumor grade and stage	Poor	^85^
	ESCC	Notch2	Upregulated	OS and PFS	Poor	^87^
	ESCC	Notch3	N/A	Chemotherapy sensitivity	Favorable	^88^
	GC	Notch1	Upregulated	Lymph node metastasis	Poor	^91^
	GC	Notch3	N/A	Immune tolerance	Favorable	^97^
	GC	JAG1	Downregulated	OS	Favorable	^98^
	PC	Notch3	Upregulated	OS	Poor	^104^
	PC	JAG1	Upregulated	OS	Poor	^106^
	PC	DLL4	N/A	Advanced tumor stage, lymph node metastasis	Poor	^107^
	CCA	Notch1	Upregulated	Tumor size, HBsAg positive	Poor	^123^
	CCA	Notch3	Upregulated	N/A	Poor	^124^
	CCA	JAG1	Upregulated	N/A	Poor	^125^
Respiratory system	NSCLC	Notch1	Upregulated	Lymph node metastasis, TNM stages	Poor	^129^
	NSCLC	Notch4, DLL4	Upregulated	Tumor size, lymph node metastasis, distant metastasis, TNM stage	Favorable	^130^
	NSCLC	JAG1, JAG2, DLL1	Downregulated	N/A	N/A	^131^
	SCLC	DLL3	Upregulated	N/A	N/A	^147^
	Postoperative SCLC	DLL3	Upregulated	PFS, chemoresistance	Poor	^148^
Hematological malignancies	Adult T-ALL	Notch1	Mutation	OS, event-free survival	Favorable	^168^
	Pediatric T-ALL	Notch1	Mutation	OS	Favorable	^169^
	CLL	Notch1	Mutation	PFS	Poor	^179^
	CLL	Notch2	Upregulated	Apoptosis characteristic	Poor	^182^
	CLL	JAG1	Upregulated	N/A	N/A	^184^
Urinary system	Bladder cancer	Notch1–3	Mutation	OS	Poor	^189^
	Bladder cancer	Notch2	Upregulated	Adverse disease parameters	Poor	^191^
	Bladder cancer	Notch3	Upregulated	Cancer-specific mortality	Poor	^193^
	Bladder cancer	JAG2	Upregulated	Tumor size, stage	Poor	^194^
	PCa	Notch1	Upregulated	High-risk, metastasis	Poor	^199^
	PCa bone metastasis	Notch3	Upregulated	Bone metastasis	Poor	^200^
	PCa	DLL3	Upregulated	Survival	Poor	^204^
	PCa	JAG1	Upregulated	Metastasis, recurrence	Poor	^201^
	RCC	Notch1	Downregulated	Tumor stage	N/A	^210^
	RCC	Notch3	Dysregulated	Chromophobe RCC, unbroken capsule, Fuhrman grade, lymph node involvement	N/A	^213^
	ccRCC	Notch	Copy number variance	OS	Favorable	^217^
	ccRCC	Notch1	Upregulated	Tumor stage, diameter	Poor	^218^
	ccRCC	JAG1	Upregulated	Tumor size, nuclear grade, TNM stage	Poor	^220^
	ccRCC	DLL4	Upregulated	Tumor grade, tumor stage, survival	Poor	^219^
Reproductive system	Breast cancer	JAG1 and Notch1	Upregulated	OS, median survival	Poor	^226^
	Breast cancer	Notch2	Dysregulated	Subgroups, genotypes	Poor	^231^
	Breast cancer	Notch4	Upregulated	Tumor size, lymph node involvement, metastasis stage	Poor	^227^
	Breast cancer	DLL3	Upregulated	Immune cell infiltration	Poor	^229^
	Breast cancer	DLL4	Upregulated	Nodal and distant metastasis	Poor	^230^
	Breast cancer	DLL4, JAG1	Upregulated	Metastasis, tumor stage	Poor	^228^
	OC	Notch1	Upregulated	Pathology stage, OS	Poor	^176^
	OC	Notch2/3, DLL3	Upregulated	Overall, disease-free survival, stages	Poor	^242^
	OC	Notch3	Upregulated	Progression-free/OS	Poor	^243^
	OC	DLL4	Upregulated	Survival	Poor	^244^
	CC	JAG1/Notch1	Upregulated	Invasion, lymph node metastasis, FIGO system	Poor	^254^
	CC	DLL4	Upregulated	Death and recurrence	Poor	^255^
Nervous system	Oligodendroglioma	Notch1	Mutation	Survival	Poor	^266^
	Glioma	Notch1	Upregulated	OS	Poor	^267^
	Glioma	Notch3	Upregulated	Grade, OS	Poor	^268^
	Glioma	DLL3	Upregulated	Prognosis	Favorable	^269^
	Neuroblastoma	Notch1	Upregulated	Advanced tumor stages, MYCN amplification, undifferentiated histology, low CRT expression level	Poor	^281^
Tumors of other systems	Melanoma	Notch4	Mutation	OS	Favorable	^293^
	Osteosarcoma	Notch3	Upregulated	Survival	Poor	^296^
	Osteosarcoma	JAG1	Upregulated	Metastasis, recurrence	Poor	^297^
	Osteosarcoma	DLL4	Upregulated	Enneking stage and metastasis, tumor differentiation	Poor	^298^
	Thyroid cancer	Notch1	80.5% positive	Lymph node metastasis	Poor	^307^
	Thyroid cancer	DLL4	54% positive	Invasion and metastasis	Poor	^308^
	Thyroid cancer	Notch3	Downregulated	Tumor size, distant metastasis, survival	Favorable	^309^
	OSCC	Notch1	Upregulated	T-stage, clinical stage	Poor	^314^
	OSCC	Notch3	33% positive	Tumor size	Poor	^316^
	OSCC	Notch4	Upregulated	Late stage	Poor	^317^
	HNSCC	Notch1/2/3	Mutation	N/A	N/A	^326^
	HNSCC	Notch1	Upregulated	Early stages, non-recurrent disease, better disease-specific, OS	Favorable	^330^

*CRC* colorectal cancer, *HCC* hepatocellular carcinoma, *ESCC* esophageal squamous cell carcinoma, *GC* gastric cancer, *PC* pancreatic cancer, *CCA* cholangiocarcinoma, *NSCLC* non-small cell lung cancer, *SCLC* small cell lung cancer, *T-ALL* T cell acute lymphoblastic leukemia, *CLL* chronic lymphocytic leukemia, *PCa* prostate cancer, *RCC* renal cell carcinoma, *ccRCC* clear cell renal cell carcinoma, *OC* ovarian cancer, *CC* cervical cancer, *OSCC* oral squamous cell carcinoma, *HNSCC* head and neck squamous cell carcinoma, *OS* overall survival, *PFS* progression-free survival, *TNM* tumor-node-metastasis

### Digestive system tumors

#### Colorectal cancer (CRC)

CRC stands out as one of the most prevalent malignant cancers globally. The expression of members from the Notch family in CRC has been extensively investigated. Numerous studies have demonstrated the high expression of Notch1 in human CRC.^56,57^ Elevated Notch1 expression has been closely associated with lymph node metastasis, tumor stage, depth of infiltration, and histological differentiation.^58^ Conversely, the expression of Notch2 in CRC was significantly negatively correlated with Notch1, and reduced Notch2 expression independently predicted a poor prognosis in CRC.^59^ Additional research has indicated that the overexpression rate of nuclear Notch3 in CRC was 38%, and nuclear Notch3 expression was closely linked to distant relapse-free survival in stage II CRC.^60^ Furthermore, the co-expression of nuclear Notch3 and Notch1 predicted a worse prognosis than negative subtypes. Regarding Notch4, researchers have verified that Notch4 expression is decreased in CRC, and the Notch4 mRNA level may serve as an independent prognostic factor for disease-free survival and overall survival (OS) in patients with CRC.^61^ Notch ligands, JAG1,^62^ JAG2,^63^ and DLL4,^64^ have also been reported to be significantly upregulated in CRC, and their high expression can predict a poor prognosis in CRC. Notably, Varga and colleagues revealed that AKT-dependent Notch3 activation led to tumor invasion and metastasis in a *Trp53*^ΔIEC^*Akt*^E17K^ mice model of mesenchymal CRC subtype, indicating that Notch3 may represent a potential target for patients with this CRC subtype.^65^ Inhibition of Notch signaling pathway by Aes, an endogenous metastasis suppressor, can block transendothelial migration and intravasation of colon cancer cells.^66^ Further study is needed to understand the role of Notch signaling in modulating the development of CRC.

#### Hepatocellular carcinoma (HCC)

The deregulated expression of Notch receptors and their ligands has been noted in HCC.^67,68^ Ahn and colleagues observed cytoplasmic expression of Notch1, Notch3, and Notch4 in 50.3%, 20.8%, and 59.7% of 288 HCC cases, respectively.^69^ Notch1 expression and Notch4 overexpression may independently predict poor survival in HCC. Another study revealed that in hepatitis B virus (HBV)-related HCC tissues, the expression of Notch1 or Notch4 was associated with HBV X protein (HBx), suggesting that HBx may play a role in carcinogenesis by regulating the Notch pathway.^70^ Notch2 is closely linked to liver cancer occurrence. Hayashi et al. found positive Notch2 nuclear staining in 19% of human primary HCC through immunohistochemistry.^71^ Consistent with this, upregulation of Notch2 was observed in human HCC cell lines.^72^ Functionally, Michael et al. found that the constitutive Notch2 signaling in the liver accelerated diethylnitrosamine-induced tumorigenesis through promoting proliferation and less differentiated HCC.^73^ Notch3 is overexpressed in HCC compared to normal liver tissue and is positively correlated with increased invasiveness and shorter survival.^74^ Another study reported abnormal accumulation of Notch3 and Notch4 in 78% and 68% of HCC tissues, respectively.^75^ JAG1 is highly expressed in HCC, with expression of JAG1 and DLL4 in HCC cells at 57.2% and 88.9%, respectively.^76,77^ However, no correlation between DLL4 expression and clinical features has been observed. Upregulated expression of JAG2 was also noted in HCC tissues and was associated with poor clinicopathological features.^78^ Targeted inhibition of JAG1 and JAG2 is expected to act as a tumor suppressor in HCC.^79^ In conclusion, these studies suggest that Notch family members may serve as potential prognostic indicators in patients with HCC.

#### Esophageal squamous cell carcinoma (ESCC)

Mutations of Notch receptors have been reported to dysregulate the Notch pathway in the development of ESCC.^80^ Li et al. identified an aberrant Notch signaling pathway in 38.3% of ESCC cases, with univariate analysis revealing an association between Notch2 gene mutations and shorter progression-free survival (PFS).^81^ Jones and colleagues found that Notch1 mutant clones were present in most human normal esophageal epithelium.^82,83^ A high proportion of biallelic mutations can block Notch1 signaling and hinder carcinogenesis, while wild-type Notch1 is conducive to the development of ESCC.^84^ Additional studies indicated that Notch1 expression in ESCC was significantly higher than in benign and reactive esophageal epithelium, showing a positive correlation with tumor grade and stage.^85^ The missense mutation site in the Notch1 gene was found to be located in the region where Notch1 binds to DLL4, enhancing the Notch1-DLL4 interaction.^86^ This may lead to resistance to neoadjuvant chemotherapy in patients with ESCC by promoting the activation of the Notch1 signaling pathway. Additionally, both mRNA and protein levels of Notch2 were significantly increased in ESCC tissues, serving as an independent predictor of poor OS and PFS.^87^ In vivo and in vitro experiments demonstrated that chemotherapy resistance in ESCC was associated with the down-regulation of Notch3 and simultaneous activation of EMT process.^88^ The ectopic expression of Notch3-activated forms inhibited EMT and increased sensitivity to chemotherapy, suggesting that Notch3 could be a potential biomarker for predicting favorable clinical outcomes in ESCC.

#### Gastric cancer (GC)

The activation of Notch signaling has been identified as a crucial factor in the development of GC. Studies have underscored the significant role of activated Notch signaling in GC development, revealing variations in the level of Notch signaling family molecules.^89,90^ Huang et al. observed higher levels of Notch1 expression in GC tissues compared to adjacent non-tumor tissues, suggesting a potential carcinogenic role for Notch1 in GC.^91^ A feedback loop between Notch1 and HGF/c-Met signaling pathways has been proposed, potentially contributing to drug resistance in GC.^92^ The high nuclear translocation frequency of Notch2 in GC (97.3%) indicates a close association between Notch2 level and GC formation.^93^ Mechanistically, both activated Notch1 and Notch2 receptors can drive GC progression through cyclooxygenase-2.^94^ Additionally, studies have reported that Notch signaling regulates the function of LGR5^+^ gastric stem cells and Cck2r^+^ antral stem cells, which is associated with gastric tumorigenesis.^95,96^ In contrast to the roles of Notch1 and Notch2, highly expressed Notch3 is implicated in the immune tolerance of GC, correlating with low infiltration of activated CD8^+^ T cells and high infiltration of immunosuppressive cells in the tumor microenvironment (TME).^97^ This suggests Notch3 could serve as a biomarker for a favorable prognosis in GC. The Notch ligand, JAG1, exhibits significantly lower levels in GC tissues than in non-tumor tissues, and its reduced level in both tumors and non-tumors is associated with poor outcomes.^98^ However, no significant difference was observed between DLL4 expression and clinicopathological features and OS.^99^ Further research is needed to explore the mechanisms underlying abnormally activated Notch signaling in GC tumorigenesis.

#### Pancreatic cancer (PC)

The Notch signaling pathway plays a crucial role in the regulation of pancreatic development and may be implicated in the differentiation, proliferation, and apoptosis of malignant pancreatic cells.^100^ Initial research indicates that Notch signaling undergoes reactivation during the initiation of pancreatic ductal adenocarcinoma (PDAC), suggesting its involvement in promoting PDAC progression.^101^ However, during the development of pancreatic intraepithelial neoplasia, Notch receptors demonstrate tumor-suppressive effects. The activated Notch pathway appears to influence the neurovascular development of PC and contributes to maintaining the population of pancreatic cancer stem cells (CSCs).^102^ In studies involving patients with PDAC, Notch3 is frequently upregulated in the cytoplasm of tumors compared to normal pancreatic ductal tissues.^103^ In patients with unresectable PC, decreased Notch3 mRNA level is significantly associated with longer OS.^104^ Inhibiting Notch3 enhances the sensitivity of PC cells to gemcitabine (GEM) chemotherapy by reducing the activity of the PI3K/AKT pathway.^105^ In both in vivo and in vitro studies, the expression of JAG1 in PC is significantly higher than that in normal pancreatic tissue.^106^ Combined treatment involving silencing JAG1 and GEM demonstrates a synergistic anti-tumor effect, suggesting that JAG1 may serve as a promising therapeutic target for PC. Furthermore, patients with PC as well as low expression of DLL4 and HES1 exhibit better survival compared to those with high expression.^107,108^ Low DLL4 abundance in tumor cells can predict the benefits of GEM adjuvant therapy after PDAC resection, and inhibiting DLL4/Notch signaling may represent a novel approach for PC therapy.^109,110^

#### Cholangiocarcinoma (CCA)

CCA, an aggressive form of biliary tract cancer with high incidence and mortality rates, can be categorized into intrahepatic (iCCA), perihilar CCA, or distal CCA based on anatomical location.^111^ Accumulating evidence indicates that the Notch pathway participates in the transformation of mature hepatocytes into malignant cholangiocytes.^112,113^ Cyclin E gene was identified as a direct transcriptional target of Notch signaling and involved in the formation of CCA caused by over-activated Notch signaling pathway.^114^ Consequently, the Notch1 pathway has been reported to mediate iCCA cell growth and the transition of the cell cycle from G0/G1 to S-phase.^115^ Notch2 is recognized as the primary determinant of iCCA formation derived from mouse hepatocytes.^116^ Mechanistically, Wang et al. uncovered that DLL4-Notch4-Efnb2 signaling mediates the differentiation of hepatic sinusoidal endothelial cells around the portal vein into apical endothelial cells, facilitating the progression of iCCA.^117^ Additionally, Hu et al. identified a novel Notch-YAP1/TEAD-DNMT1 axis that drives hepatocyte reprogramming into iCCA.^118^ Another study revealed that elevated fucosylation is a hallmark of human iCCA, promoting cell growth and migration by upregulating Notch and EGFR/NF-κB pathways.^119^ Simultaneously, the Notch pathway is considered a key indicator of CCA progression and prognosis.^120,121^ Studies show that Notch1 is upregulated in iCCA, potentially promoting iCCA migration by inducing EMT.^122,123^ Additionally, Guest et al. identified the differential overactivation of the atypical receptor Notch3 in iCCA in humans, rats, and mice.^124^ Notch3 activates the PI3K/AKT cascade through a non-classical pathway, maintaining tumor cell survival. Che et al. found that JAG1 is generally up-regulated in human iCCA samples compared with non-neoplastic livers, and inhibiting JAG1 can increase the apoptosis of human iCCA cell lines.^125^ Importantly, JAG1 is a crucial upstream inducer of Notch signaling in human and mouse iCCA.^125^ The synergistic overexpression of JAG1 and activated AKT signaling promotes the occurrence of liver cancer.^125^ In summary, activated Notch signaling is identified as a common carcinogenic event in human CCA. A deeper understanding of the mechanisms triggered by the Notch pathway and its functional crosstalk with other signaling cascades may contribute to the design of new therapies for human CCA.

### Respiratory system tumors

#### Non-small cell lung cancer (NSCLC)

Lung cancer stands as one of the most lethal cancers globally, contributing to ~25% of all tumor-related fatalities.^126^ Based on histopathological features, lung cancer is broadly categorized into two major types: NSCLC and small cell lung cancer (SCLC), with NSCLC encompassing 80–85% of all lung cancer cases.^127^ NSCLC further differentiates into two primary subtypes: lung adenocarcinoma (LUAD) and LUSC. Unfortunately, more than half of patients with NSCLC receive a diagnosis at an advanced disease stage, and the efficacy of combination chemotherapy hovers at ~20%. Consequently, comprehending the pathogenesis of NSCLC and overcoming chemotherapy resistance is pivotal to enhancing the prognosis of NSCLC.

Over the decades, the Notch signaling pathway has garnered increasing attention as a promising new target for diagnosing and prognosing NSCLC. Notch1 is detected in 50% of stage I to IV NSCLC cases, predominantly localized to the cell membrane and cytoplasm.^128^ Meta-analysis reveals that high Notch1 expression correlates positively with lymph node metastasis and higher tumor-node-metastasis (TNM) stage, indicative of poor OS in patients with NSCLC.^129^ Wang et al. identified significantly higher positive rates of Notch4 and DLL4 in NSCLC compared to normal lung tissues.^130^ However, contradictory findings emerge from another study, reporting downregulated DLL4 protein levels in NSCLC tissues and lung cancer cell lines. The levels of other Notch ligands, including JAG1, JAG2, and DLL1, in NSCLC were also observed to be lower than that in normal lung tissue.^131^ Furthermore, DLL4 and Notch1 emerged as independent prognostic factors for NSCLC but exhibited varying effects in LUAD and LUSC.^132^ The inconsistent results across studies may stem from small sample sizes or variations in sample sources. A comprehensive, large-scale, multi-center study is imperative to thoroughly investigate the expression and function of Notch family members in NSCLC.

Accumulating evidence suggests that the evolutionarily-conserved Notch signaling pathway plays a crucial role in cell specification and fate determination during lung development, and it also mediates the initiation and progression of NSCLC.^133^ For instance, Xie et al. discovered that Notch1 contributes to the EMT phenotype of NSCLC, promoting acquired resistance in NSCLC.^134^ Another study demonstrated that the activated Notch1 signal forms a positive feedback loop with the downstream functional transcription target RFC4, conferring metastasis and stemness characteristics to NSCLC cells, as well as resistance to γ-secretase inhibitor (GSI) treatment.^135^ Furthermore, Baumgart and colleagues established that Notch signaling regulates tumorigenesis in Kras^G12D^-driven LUAD.^136^ Surprisingly, Notch1 and Notch2 play distinct roles in NSCLC, where Notch2 mediates differentiation and inhibits tumor formation during lung cancer progression, while Notch1 promotes carcinogenesis. Notably, Zheng et al. identified a rare population of CD24^+^ITGB4^+^Notch^hi^ cells from a Kras-driven NSCLC mouse model, which drives tumor progression, and Notch3 has a specific and non-redundant function in mediating the propagation and self-renewal of tumor-propagating cells.^137^ Importantly, a co-expression analysis revealed that Notch1 exhibits opposite functional effects on angiogenesis and immune pathways in LUAD and LUSC, potentially contributing to the development of Notch1-dependent targeted therapy strategies for specific tumor subgroups within NSCLC.^138^

#### Small cell lung cancer

SCLC is a high-grade neuroendocrine cancer that constitutes 13–5% of newly diagnosed lung cancers, with a daunting five-year survival rate of less than 7%.^139,140^ This aggressive cancer is characterized by high genomic instability, rapid growth, and a substantial potential for metastasis.^141^ Over 60% of patients with SCLC are diagnosed with extensive-stage SCLC, facing a median survival of less than 10 months.^142^ Even for those diagnosed with limited-stage SCLC, survival rates are generally poor. While initial responses to standard chemotherapy and radiotherapy are common, rapid relapse due to acquired chemotherapy resistance is a frequent challenge. The uncommon preinvasive histological pattern of SCLC makes traditional early screening strategies ineffective. Therefore, a deeper understanding of SCLC biology, the development of novel predictive biomarkers, and the search for new therapeutic targets are crucial for improving SCLC prognosis.

Dysregulated gene expression patterns and activity of the Notch family have been identified in SCLC. Interestingly, the frequency of gene mutations in the Notch signaling pathway among Chinese patients with SCLC is significantly lower compared to Western populations.^143^ Almodovar and colleagues reported that 52% of patients with SCLC exhibit inactivating mutations of Notch family genes in their plasma cell-free DNA.^144^ Another study found that 20–25% of SCLC cases carry loss-of-function Notch mutations.^145,146^ The cell surface protein DLL3, highly selective for tumors, is expressed in 85% of patients with SCLC.^147^ Notably, DLL3 expression remains robust across all stages of SCLC and remains stable despite therapeutic interventions. In a study involving postoperative patients with SCLC treated with platinum and etoposide plus anti-programmed cell death ligand 1 antibody, it was observed that SCLC with high DLL3 expression developed resistance to immunochemotherapy due to tumor immunosuppression, despite having a higher load of neoantigens.^148^ Functionally, DLL3 acts as a regulator of cell-cell interactions in the neuroendocrine state of SCLC.^149^ Numerous preclinical and clinical studies targeting DLL3 are underway, defining it as a promising treatment strategy for SCLC.^150,151^

With the exploration of molecular aberrations in SCLC, dysregulation of the Notch pathway has emerged as one of the driving factors in tumorigenesis and intratumoral heterogeneity in SCLC. Activated Notch signaling induces profound G1 cell cycle growth arrest and significantly decreases neoplastic potential. SCLC displays a high degree of heterogeneity, with multiple subtypes coexisting within individual tumors, exhibiting both neuroendocrine cell characteristics and non-neuroendocrine phenotypes in both mouse and human SCLC tumors.^152^ Ireland et al. demonstrated that MYC mediates the neuroendocrine plasticity of SCLC through the activation of Notch signaling.^153^ Specifically, endogenous activation of the Notch pathway leads to a fate switch from neuroendocrine to non-neuroendocrine in 10–50% of SCLC cells.^154^ Notch signaling plays a dual role in SCLC, acting as a tumor suppressor in neuroendocrine cells and as a driver of increased chemoresistance in non-neuroendocrine cells to support SCLC growth.^155^ Importantly, in preclinical models, the combination of Notch inhibition and chemotherapy effectively suppresses SCLC tumor growth and the generation of non-neuroendocrine cells. The recognition that the Notch pathway initiates tumor heterogeneity and treatment resistance in SCLC has inspired the development of personalized treatment strategies targeting Notch signaling for different SCLC subtypes.

### Hematological malignancies

#### T cell acute lymphoblastic leukemia

T-ALL is an aggressive hematological malignancy, constituting 15% and 25% of ALL cases in children and adults, respectively, with a high recurrence rate and poor prognosis. This malignancy is characterized by acquired chromosomal translocations and genetic alterations, resulting in aberrant expression of transcriptional regulators.^156,157^ Notch signaling through Notch1 receptors is crucial for T cell lineage development, thymocyte survival, and proliferation of committed T cell progenitors.^158,159^ A seminal study has identified Notch1-activated point mutations in over 50% of T-ALL cases, underscoring the prominent role of Notch1 signaling cascades in T-ALL pathogenesis.^160^ For instance, in Ikaros-deficient T-ALL, T cell-specific deletion of floxed Notch1 promoter/exon 1 sequences promotes the activation of oncogenes and accelerates leukemia onset.^161^ Another study revealed that overexpression of intracellular Notch1 in hematopoietic progenitor cells leads to abnormal lymphatic development, crucial for tumor maintenance.^162^ Furthermore, abnormal expression of CD44 serves as an early marker of mutant Notch1 signaling and extrathymic T cell development, suggesting that Notch1 signaling may contribute to T-ALL pathogenesis by inducing CD44 expression.^163^

The presence of Notch1 mutations in patients with T-ALL raises questions regarding the prognostic impact of Notch signaling alterations. F-box and WD40 repeat domain containing-7 (FBXW7), an E3 ubiquitin ligase, has been reported to recognize and bind to the Notch1 PEST domain, leading to degradation of the activated form of Notch1.^164,165^ FBXW7 mutations stabilize intracellular Notch1 in the nucleus and are thought to work synergistically with the Notch1 PEST mutations.^166,167^ An early study involving 141 adult T-ALL samples identified 62% with Notch1 mutations and 24% with FBXW7 mutations. The study suggested that activation of the Notch1 pathway due to Notch1/FBXW7 mutations could identify patients with a favorable prognosis.^168^ Among 162 treated pediatric patients with T-ALL screened in the MRC UKALL2003 trial, those with double mutations of Notch1 and/or FBXW7 exhibited very positive outcomes.^169^ Overall, these studies indicate that Notch activation may be associated with improved early treatment response in T-ALL, and the impact on prognosis may be influenced by differences in treatment approaches.

#### Chronic lymphocytic leukemia

CLL is characterized by the expansion of monoclonal CD5^+^CD23^+^ B cells in peripheral blood, bone marrow, and secondary lymphoid tissues.^170^ CLL has a genetic susceptibility, with family members of patients with CLL having a 6–9 times increased risk.^171^ Recent advancements have unraveled the genetic landscape of CLL, exposing genomic heterogeneity among different patients with CLL.^172,173^ Approximately 10% of CLL cases carry Notch1 gene mutations at diagnosis.^174,175^ These mutations, located in the coding region or 3’ untranslated non-coding regions of the Notch1 gene, result in impaired degradation and accumulation of the Notch1 intracellular domain (N1ICD).^176^ CLL cells with Notch1 mutations display partial chemotherapy resistance in vitro, indicating that Notch1 could be a potential molecular target for CLL. Another study revealed that Notch1 mutations in CLL are associated with relative resistance to low CD20 expression and in vitro anti-CD20 immunotherapy, suggesting epigenetic dysregulation of CD20 expression mediated by histone deacetylases.^177^ A retrospective analysis of 317 Chinese patients with CLL identified Notch1 mutation as an unfavorable prognostic factor.^178^ Consistent results were observed in a prospective multicenter COMPLEMENT1 trial, linking Notch1 mutations to poor PFS.^179^ Further research is needed to explore the molecular mechanisms of Notch1 mutations, their impact on prognosis, and suitable strategies for treating patients with CLL with Notch1 mutations.

Abnormal Notch signaling accelerates the proliferation of CLL cells and contributes to disease progression.^180,181^ In B-cell CLL cells, the oncogenic gene Notch2 is highly expressed and associated with disease-specific apoptosis failure.^182^ Notch2 high expression characterizes a subset of patients with CLL, mainly carrying trisomy 12, which is marked by high levels of Mcl-1.^183^ Silencing Notch2 to reduce Mcl-1 expression can restore the response of CLL cells to venetoclax treatment. Additionally, Filomena et al. provided evidence that JAG1 is constitutively processed in CLL cells, and the activation of Notch1/2 is independent of the up-regulation of JAG1 levels.^184^ These findings offer new insights into Notch signaling in CLL cells and suggest that targeting the Notch signaling pathway could be developed as a novel therapeutic strategy for CLL.

### Urinary system tumors

#### Bladder cancer

According to the World Health Organization, nearly 600,000 people are diagnosed with bladder cancer each year, with smoking and workplace exposure to suspected carcinogens being the main risk factors for bladder cancer.^185,186^ Bladder cancer is three to four times more common in men than in women.^187^ However, women are often diagnosed with advanced disease at the onset and have a poorer prognosis. Currently, there is a lack of ideal treatment methods for bladder cancer. Therefore, exploring the molecular mechanisms of bladder cancer and identifying early diagnosis and treatment targets holds promise for improving the prognosis of patients with bladder cancer. In recent years, the disparate roles of Notch signaling in bladder cancer have been established, with its oncogenic and tumor -suppressive effects depending on tissue type and cellular environment.^188^ Rampias et al. reported that more than 40% of human bladder cancers carry new inactivation mutations of Notch pathway components.^189^ Moreover, they found that activated Notch inhibits the proliferation of bladder cancer cells, indicating that the loss of Notch activity is a driver of urothelial carcinoma. Similarly, Maraver et al. revealed that missense mutations in Notch1 and Notch2 in human bladder cancer lead to functional loss of the Notch pathway, favoring the EMT process and promoting the aggressive character of bladder cancer.^190^

Overall, considerable research supports the function of Notch1 as a tumor suppressor in bladder cancer. In contrast, further work demonstrated that Notch2 functions as an oncogene. Hayashi et al. revealed a high incidence of increased Notch2 copy number in bladder cancer, and Notch2 activation is associated with a poorer prognosis.^191^ Additionally, the Notch2/HEY1 signaling pathway mediates cancer-associated fibroblasts (CAFs)-derived microfibrillar-associated protein 5 to promote the proliferation and metastasis of bladder cancer.^192^ In the case of Notch3, a study involving 614 urothelial bladder cancer samples showed that 91.5% of samples expressed Notch3, and the degree of positive Notch3 expression was positively associated with the risk of cancer-specific death.^193^ Moreover, the gene expression and protein levels of JAG2 were reported to be progressively up-regulated with the increase in primary tumor size and histopathological stage.^194^ Together, these findings provide evidence that Notch signaling has a dual role in bladder cancer. However, many unsolved problems about the mechanism of Notch signaling in bladder cancer still need further study in the future.

#### Prostate cancer (PCa)

PCa is the second most common cancer in men, with more than 1.2 million newly diagnosed cases worldwide each year.^195^ PCa is highly heterogeneous, and its progression can be driven by gene mutations and DNA damage response.^196^ Although the long-term survival rate of local PCa is satisfactory, metastatic PCa is largely incurable even after intensive comprehensive treatment.^197^ Over the decades, extensive evidence has suggested that Notch signaling is involved in prostate development and the maintenance of adult prostate homeostasis. Abnormal expression of Notch receptors and ligands leads to Notch signaling dysfunction, which regulates tumor formation and progression in PCa as an oncogene or tumor suppressor gene.^198^ For example, previous studies have shown that local high-risk PCa and metastatic castration-resistant PCa cells express high levels of Notch1 receptors, and activated Notch1 cooperates with multiple carcinogenic pathways to drive the invasiveness of PCa.^199^

PCa metastasis primarily occurs in the bone, where it induces a unique osteoblastic response. Studies have found that Notch3 expression is elevated in human PCa bone metastasis.^200^ Notch3 inhibits osteoclasts and stimulates osteoblastogenesis by inducing MMP-3, thereby promoting osteoblast bone metastasis. A study involving 154 PCa samples indicated that JAG1 expression is higher in metastatic PCa than in localized PCa or benign prostate tissue.^201^ Additionally, high expression of JAG1 in clinically localized tumors is apparently related to recurrence. Mechanistically, in the phosphatase and tensin homolog (PTEN)-deficient PCa mouse model, overexpression of JAG1 can up-regulate transforming growth factor-beta (TGF-β) signaling in prostate stromal cells and promote the formation of a reactive matrix microenvironment.^202^ In addition, Tran et al. found that overexpression of JAG1 intracellular domain (JAG1-ICD) enhances the stem-like characteristics and mobility of PCa cells.^203^ In patients with advanced metastatic PCa, Chou et al. revealed that DLL3 is expressed in de novo and advanced small cell/neuroendocrine carcinoma (SCNC) PCa, and is associated with poor survival rates.^204^ Immunotherapy targeting DLL3 showed anti-tumor potential in invasive SCNC. Although a large number of studies based on clinical PCa samples, cancer cell lines, and animal models have suggested that Notch pathway elements are dysregulated in PCa, the function of Notch signaling in PCa is still not fully determined.^205,206^ Based on the current knowledge, more sufficient research is still needed to provide reliable evidence for targeting the Notch signaling pathway in the treatment of PCa.

#### Renal cell carcinoma (RCC)

RCC is the most common malignancy of the genitourinary system, with a mortality rate of 30–40%.^207^ Previous studies have shown that several key molecules of the Notch cascade are expressed during nephrogenesis, and dysregulated Notch activity may play a vital role in the development of RCC.^208,209^ Sun et al. observed that the expression of Notch1 and Notch4 in RCC was either absent or significantly down-regulated compared with adjacent non-tumor tissues.^210^ Functionally, HES1-mediated down-regulation of microRNA miR-138 maintains the activation of the Notch1 pathway and facilitates the malignant progression of RCC.^211^ Consistently, selective Notch1 suppression by small interfering RNA could inhibit RCC cell proliferation via the JNK/p38 pathway.^212^ Another study revealed that Notch3 was positively correlated with chromophobe RCC, unbroken capsule, Fuhrman grade 1, and less lymph node involvement.^213^ Wang et al. found that DLL4 may participate in the development of RCC by engaging in signal transduction and angiogenesis.^214^ Blocking DLL4 showed effective antitumor activity in RCC patient-derived xenografts.^215^ Together, these studies suggest that the Notch pathway may represent previously overlooked treatment opportunities for RCC.

Clear cell RCC (ccRCC) is the most common histological subtype of RCC, accounting for ~75% of all kidney cancers.^216^ ccRCC is characterized by heterogeneity and potential genetic predisposition. A study involving 415 patients with ccRCC found that 44% of Notch genes had genetic alterations, with copy number variation being the main type of gene variation.^217^ Additionally, patients with ccRCC with Notch pathway alterations had better OS. Another study found that the expression of Notch1 and JAG1 in ccRCC tissues was higher than in normal adjacent tissues.^218^ The upregulation of Notch1 signaling promotes the proliferation and migration of tumor cells, increasing the risk of metastasis in T1 stage ccRCC. In addition, the expression of DLL4 and JAG1 in ccRCC were significantly higher than those in normal renal tissues and were positively correlated with poor prognosis.^219,220^

### Reproductive system tumors

#### Breast cancer

Breast cancer is the most commonly diagnosed malignancy in women, accounting for 31% of all female cancers.^221^ For nearly half a century, the incidence of breast cancer has continued to rise.^222^ Although the development of surgery, radiotherapy, chemotherapy, endocrine therapy, and targeted therapy has improved the 10-year survival rate of breast cancer, 30–40% of patients still face significant challenges of metastasis and recurrence. Over the years, Notch has been implicated as a contributor to breast cancer, potentially due to its role in breast cancer stem-like cell (CSLC) characteristics, EMT, resistance to chemotherapy, and other processes.^223–225^ Studies have revealed that highly expressed Notch1, Notch4, JAG1, DLL3, and DLL4 are observed in breast cancer with poor prognosis, suggesting that Notch signaling is promising as a biomarker for breast cancer prognosis.^226–230^ The expression of Notch2 in rs11249433 risk genotype (AG/GG) carriers was significantly increased, which may promote the development of estrogen receptor-positive luminal breast cancer.^231^ Functionally, Notch signaling activates aldehyde dehydrogenase 1A1 (ALDH1A1) by inducing Sirtuin-2, resulting in ALDH1A1 deacetylation and enzymatic catalysis, accelerating breast CSCs.^232^ Claudin-low breast cancer is thought to originate from breast stem cells, characterized by stemness and an EMT phenotype. Zhang et al. reported that Notch mediates Manic Fringe-induced PIK3CG transcription, promoting the Claudin-low breast cancer phenotype.^233^ As early as 2006, Myc gene was identified as a direct transcriptional target of Notch1 and a necessary factor for Notch1-induced breast tumorigenesis in mice.^234^ Besides, Notch1 activation promotes triple-negative breast cancer (TNBC) formation by initiating ATR-CHK1 signaling cascade, restoring S/G2 and G2/M cell cycle checkpoints, and inhibiting mitotic catastrophe.^235^ Additionally, Notch signaling regulates the EMT process of breast cancer cells through various mechanisms, such as a Slug-dependent manner, the S100A16/Notch1 pathway, the FYN/STAT5 pathway, and the Notch4/STAT3 signaling pathway.^236,237^ Notch ligands have been proven to play an important role in breast cancer drug resistance. Collectively, Notch signaling plays a carcinogenic function in breast cancer.^238,239^ Robinson and colleagues identified functionally recurrent rearrangements of Notch gene families in breast cancer, with certain therapeutic implications.^240^ Given the complexity of the Notch pathway, further exploration is needed to develop successful Notch targeting strategies to prevent and treat breast cancer.

#### Ovarian cancer (OC)

OC is one of the leading causes of cancer death in women, with a five-year survival rate of ~50% and an even worse prognosis for metastatic disease.^241^ Early identification of high-risk women for OC is crucial due to the predominance of nonspecific symptoms occurring in the late clinical stages. In recent years, Notch signaling has been increasingly studied in OC, which may be used as a biomarker to predict prognosis. A study of 328 patients with primary OC revealed that high expression of N1ICD in female OC is an independent risk factor for poor prognosis.^176^ Consistent results were obtained through large data portals, suggesting that upregulation of Notch signaling family proteins in OC is generally related to poorer survival and more advanced cancer stages.^242^ Another study observed that Notch3 overexpression was related to shorter survival in patients with advanced OC treated with platinum and taxane.^243^ DLL4 was found to be overexpressed in 72% of OC, which is an independent predictor of poor survival.^244^

Many studies have linked Notch signaling components to the malignant characteristics of OC. JAG1 promotes the EMT process of OC by crosstalk with the JAK/STAT3 pathway, further enhancing the invasion and migration ability of platinum-resistant OC.^245^ In OC, tumor-associated neutrophils activate JAG2 to coordinate the intratumoral IL-8-driven immune evasion microenvironment.^246^ Resistance to standard treatment regimens is one of the main reasons for the poor prognosis of OC. The Notch1 signaling pathway mediates paclitaxel (PTX) resistance in CD44^+^CD117^+^ OC cells promoted by chemokine CCL20.^247^ The activated Notch3 pathway mediates the nuclear receptor NR2F6 to promote epithelial OC (EOC) cells’ resistance to cisplatin.^248^ Similarly, Notch3 enhances EMT in OC cells and attenuates carboplatin-induced apoptosis.^249^ Together, these studies elucidate the molecular mechanisms by which the Notch signaling pathway contributes to OC aggressiveness and chemotherapy resistance in vivo and in vitro.

#### Cervical cancer (CC)

CC is a major public health problem affecting middle-aged women. From 2001 to 2019, 227,062 new cases of CC were reported in the United States.^250^ There is conclusive evidence that the high-risk subtype of human papillomavirus (HPV) infection is a leading cause of CC.^251^ Talora et al. reported that the specific down-regulation of Notch1 signaling in CC cells leads to the continuous transcription of HPV-driven E6/E7 viral genes and plays a key role in HPV-induced advanced carcinogenesis.^252^ In turn, the activated Notch1 signaling inhibits the activity of E47 through the RBP-Jk-dependent mechanism, inducing the growth arrest of HPV-positive CC cells.^253^ However, Yousif et al. found that Notch1 and JAG1 were overexpressed in CC and were associated with poor OS.^254^ Another study showed that HPV16 E6 could induce the continuous expression of DLL4 in keratinocytes, and the high expression of DLL4 was closely related to the poor prognosis of CC.^255^ The role of Notch signaling in CC is complex.^256,257^ CD66^+^ cells in primary invasive CC exhibit high Notch signaling and tumorigenicity.^258^ Activation of Notch signaling can induce cell cycle arrest in human CC cells.^259,260^ In addition, several research studies have shown that inhibition of Notch signaling can strengthen the sensitivity of CC cells to chemotherapeutic drugs.^261^ These studies have strengthened the view that dysregulated Notch signaling is associated with the progression of CC and laid the foundation for a detailed exploration of targeted therapy.

### Nervous system neoplasms

#### Glioma

Glioma is a malignant brain tumor derived from glial progenitor cells, accounting for 80.8% of primary central nervous system tumors, resulting in serious morbidity and mortality.^262^ Previous researches have analyzed the integrated genomic characteristics of gliomas. Notch1 mutations were identified in diffuse lower-grade gliomas, Notch1 and Notch2 mutations were identified in IDH1-mutant gliomas, and grade II and III gliomas carried Notch1–4 mutations.^263–265^ Furthermore, Halani et al. uncovered that Notch1 mutations were related to disease progression and shorter survival in oligodendroglioma.^266^ Accumulating studies have highlighted the importance of the Notch signaling pathway in glioma malignancy. Recent research revealed that the expression of Notch1 and Notch3 was significantly increased in glioblastoma and promoted tumor growth activity through the NF-κB pathway.^267,268^ In addition, DLL3 was found to be up-regulated in IDH1 mutant gliomas and was associated with a better prognosis.^269^ Overall, the differential expression pattern of Notch1–4 receptors can be used as a marker of glioma differentiation and a possible prognostic factor.^270^ Functionally, Notch signaling is involved in glioma progression through complex mechanisms. For example, Notch signaling mediates miR-33a-driven self-renewal of glioma-initiating cells.^271^ Silencing Notch1 can induce autophagy and down-regulate the Notch1/HES1 pathway to inhibit the proliferation of glioma cells.^272^ Moreover, a large amount of evidence suggests that Notch signaling is involved in maintaining the characteristics of glioma stem cells (GSCs).^273^ However, Parmigiani et al. found that inhibition of Notch signaling can make proneural glioma cells evade immune monitoring and increase invasiveness.^274^ In summary, the Notch signaling pathway is heavily involved in the fate determination of glioma cells, which is related to the progression of gliomas. Targeting the Notch pathway may intervene in these processes and potentially bring better therapeutic effects for patients with glioma.

#### Neuroblastoma

Neuroblastoma is a neuroendocrine tumor originating from the sympathetic nervous system, characterized by genetic, morphological, and clinical heterogeneity.^275^ Advances in high-throughput technology have contributed to understanding the genetic changes and molecular pathways involved in the pathogenesis of neuroblastoma, including MYCN amplification, PHOX2B mutation, the PI3K/AKT/mTOR pathway, and Notch signaling. Previous studies have shown that Phox2B can control the expression of Delta-Notch pathway genes by regulating HASH1.^276^ Activation of the Notch signaling pathway leads to growth arrest in neuroblastoma.^277^ Approximately 20% of neuroblastomas carry MYCN oncogene amplification, which is related to decreased expression of genes encoding gamma-secretase subunits and Notch signaling components.^278^ In the MYCN transgenic neuroblastoma model, Notch2 signaling mediates Midkine to promote the formation and occurrence of neuroblastoma.^279^ Axelson’s study demonstrated that the Notch signaling cascade regulates HASH-1/HES-1 to participate in the differentiation of neuroblastoma cells and regulate malignant phenotypes.^280^ Hooper et al. revealed the presence of N1ICD in the sub-nuclear bodies and primary cortical neurons of SH-SY5Y neuroblastoma. High expression of Notch1 in neuroblastoma indicates a poor prognosis and is expected to be a therapeutic target for patients with neuroblastoma.^281^ Additionally, the Notch3 feed-forward loop drives the transcriptional reprogramming of neuroblastoma from adrenergic to mesenchymal states.^282^ Notch3 endows neuroblastoma cells with a highly motile phenotype, and the subpopulation with high expression of Notch3 and its downstream regulatory genes has mesenchymal characteristics, making it prone to metastasis and associated with a worse prognosis.^283^ These findings reveal the molecular mechanism of Notch signaling in neuroblastoma, which are of strategic significance for improving drug treatments in this cancer type.

### Tumors of other systems

#### Melanoma

Melanoma, the most aggressive form of skin cancer, poses a significant global public health challenge.^284^ It is estimated that, by 2020, there were a total of 325,000 new cases of melanoma worldwide, resulting in 57,000 deaths.^285^ If the incidence rate continues at the 2020 level, there is projected to be a ~50% increase in new cases of melanoma and a 68% increase in deaths by 2040. Notch signaling is believed to play a dual role as both oncogenes and tumor suppressor genes in melanoma.^286,287^ Overexpressed Notch1 signaling promotes melanoma-induced immunosuppression by upregulating TGF-β1.^288^ Additionally, in vitro studies have revealed that Notch1 signaling in CAFs acts as a molecular switch, reversing the plasticity and stemness of CSCs, thus regulating the heterogeneity and invasiveness of melanoma cells.^289^ Similarly, miR-146a-5p is transferred to astrocytes via extracellular vesicles, down-regulating NUMB and activating the Notch pathway, thereby promoting melanoma brain metastasis.^290^ Conversely, in the context of PTEN deficiency, Notch1 and Notch2 exhibit anti-tumor effects in BRAFV600E/PTEN-null-driven melanoma genesis.^291^ Likewise, Rad et al. reported that Notch4 acts as a tumor suppressor in melanoma.^292^ In NRAS wild-type melanoma, tumors with Notch4 mutations exhibit a higher tumor mutation burden and tumor neoantigen burden.^293^ Notch4-mutant tumors enhance anti-tumor immunity, resulting in a better immune therapy response and prognosis. According to the aforementioned studies, the function of Notch signaling in melanoma is highly dependent on the environment, and detailed investigations are still required to elucidate the relevant molecular mechanisms.

#### Osteosarcoma

Osteosarcoma stands as the most prevalent primary bone malignancy, demonstrating high heterogeneity and primarily affecting children, adolescents, and young adults.^294^ Despite significant advancements in chemotherapy and surgery, the survival rate of patients with osteosarcoma has shown no improvement in recent decades.^295^ Studies have revealed that molecules from the Notch signaling family are consistently overexpressed in the majority of clinical osteosarcoma samples, correlating positively with recurrence, metastasis, and poor prognosis.^296–298^ Both in vivo and in vitro experimental investigations have indicated that Notch signaling plays a crucial role in regulating the cell cycle of osteosarcoma, influencing its recurrence, lung metastasis, and malignant progression.^299,300^ Furthermore, the up-regulation of JAG1 expression has been linked to promoting the stem-like phenotype and tumor growth of osteosarcoma.^301^ Conversely, Notch signaling has been found to modulate the sensitivity of osteosarcoma to chemotherapy resistance.^302,303^ In summary, our current understanding of the intricate function of Notch in osteosarcoma is just scratching the surface, and further comprehensive research holds the potential to facilitate its clinical transformation in tumor therapy.

#### Thyroid cancer

Thyroid cancer is a prevalent malignancy within the endocrine system, comprising four primary histological subtypes: papillary thyroid cancer (PTC), follicular thyroid cancer (FTC), medullary thyroid cancer (MTC), and anaplastic thyroid cancer.^304^ The Notch receptor and ligand family have been identified as regulators of tumorigenesis in thyroid cancer.^305^ Notably, Simonetta et al. observed that the expression pattern of Notch1 varies across different histological types of thyroid cancer.^306^ Specifically, Notch1 positivity is predominantly limited to papillary carcinoma, rarely detected in follicular carcinoma and medullary carcinoma. Positive expressions of Notch1 and DLL4 were identified in PTC, showing a significant correlation with tumor invasion, metastasis, and poor prognosis.^307,308^ Conversely, the expression of Notch3 decreased in FTC specimens exhibiting reduced differentiation and increased malignancy, linking to clinicopathological features associated with poor prognosis.^309^

MTC represents a distinct type of neuroendocrine tumor originating from thyroid C cells. In MTC cells, the role of Notch signaling differs from its function in PTC cells.^310^ Muthusamy et al. confirmed that overexpression of N1ICD inhibited MTC cell proliferation and altered the neuroendocrine phenotype of MTC cells.^311^ Similarly, Notch2 also mediated cell apoptosis and inhibited neuroendocrine markers in MTC.^312^ Despite the limited and at times contradictory nature of research on the Notch pathway in thyroid cancer thus far, in-depth investigations specific to different histological subtypes of thyroid cancer are deemed necessary to elucidate the inconsistent functions of Notch in thyroid cancer comprehensively.

#### Oral squamous cell carcinoma (OSCC)

OSCC is the most prevalent oral malignant tumor worldwide, with its poor prognosis primarily attributed to metastasis and recurrence.^313^ Early diagnosis holds the potential to positively impact the survival rates of OSCC. Research indicates that Notch receptors, specifically Notch1,^314,315^ Notch3,^316^ and Notch4,^317,318^ exhibit high levels in human OSCC tissues and are associated with a poor prognosis. This suggests that Notch receptors could serve as biomarkers for the early diagnosis of OSCC. Functionally, the activated Notch-HES1 signaling pathway plays a crucial role in mediating the stem-like phenotype of OSCC and actively contributes to the progression of the disease.^319^ Furthermore, the up-regulation of Notch signaling demonstrates carcinogenic properties, promoting the proliferation, migration, and invasion of OSCC cells, thereby contributing to the malignant characteristics of OSCC.^320,321^ Importantly, Notch signaling also interacts with other cell signaling pathways, such as Wnt and Hedgehog, intensifying the aggressiveness of OSCC.^322^ A comprehensive understanding of the molecular mechanisms underlying Notch signaling in OSCC is imperative for the development of targeted therapeutic strategies aimed at tackling this challenging oral malignancy.

#### Head and neck squamous cell carcinoma (HNSCC)

HNSCC encompasses a diverse group of tumors originating from the squamous epithelium of the oral cavity, pharynx, and larynx.^323^ Over the past half-century, there has been a decline in the incidence of smoking-related HNSCC, while cases induced by HPV infection have seen a gradual increase.^324^ Studies focusing on the long tail genes of HNSCC have revealed that 67% of carcinogenic mutations in human HNSCC cases converge on Notch signaling, establishing Notch inactivation as a marker for HNSCC.^325–327^ This aligns with the recognized role of Notch1 as a tumor suppressor in HNSCC. Genomic analysis has indicated a significant mutation rate in Notch1, ranking it as the gene with the second-highest mutation frequency after TP53.^328,329^ In HNSCC tissues, Notch1 is highly expressed compared to normal tissues and is associated with a favorable prognosis.^330,331^ Targeting hypoxia-inducible factor 1 alpha (HIF1α)/Notch1 signaling has been found to mitigate the stem-like characteristics and chemotherapy resistance of HNSCC CD44^+^ cells.^332^ Despite these insights, our understanding of the intricate functions of Notch signaling in HNSCC remains at a preliminary stage.^333,334^ Elucidating how Notch signaling acts as either an oncogene or a tumor suppressor at different stages of tumorigenesis holds the key to developing new drug targets for HNSCC.

## The mechanism of Notch signaling patyway-mediated tumorigenesis and progression

Over the past two decades, extensive investigations have revealed that the Notch signaling pathway is intricately involved in various facets of cancer biology.^335–337^ This includes its role in EMT, angiogenesis, the acquisition of CSLC properties, metabolic reprogramming, regulation of the TME, and mediation of chemotherapy resistance. Dysregulation of Notch signaling can function either as an oncogene or a tumor suppressor, exerting influence over the progression of tumors. In the following section, we have compiled and emphasized the molecular mechanisms underlying Notch signaling-mediated tumorigenesis and progression (Table 2). Our aim is to offer new insights into potential targeted therapies for various types of cancers.

**Table 2:** Mechanisms of Notch signaling pathway-mediated tumorigenesis and progression

Cancer	Notch signaling component	Role	Involved biological process	Mechanism	Related molecule	Cell line	Ref.
ESCC	Notch1, Notch3	Oncogene	EMT	TGF-β→active Zeb1→represses Notch3→limiting terminal differentiation; TGF-β→active Notch1→drive EMT→promote SCC tumor initiation	TGF-β, ZEB1	TE11, EN60, EPC2T	^346^
HCC	Notch1	Oncogene	EMT	Tspan5 ↑ →Activation of Notch signaling→enhance EMT and actin skeleton rearrangement→promote tumor metastasis	Tspan5	HL7702, BEL7402, Hep3B, HUH7, MHCC97H, MHCC97L, PLC, QGY7701, SK-Hep1	^345^
SCLC	Notch2, HES1	Oncogene	EMT	ZLDI-8→inhibit the Notch signaling→inhibit migration, invasion and EMT phenotype of drug-resistant lung cancer	ZLDI-8	A549 cells, resistance A549/Taxol cells	^354^
Glioblastoma	RBPJ	Oncogene	EMT, chemoresistance	RBPJ ↓ →blocks EMT activators→reduce cellular invasion and resistance	ZEB1	GBM1, BTSC407/407p, JHH520/JHH	^353^
HNSCC	Notch4, HEY1	Oncogene	EMT	Notch4-HEY1 pathway ↑ →promote EMT→induce proliferation and cisplatin resistance	E-cadherin, Vimentin, Fibronectin, TWIST1, and SOX2	SKN3, Cal27, SCC61, and SCC090	^347^
ACC	HEY1, Notch1	Oncogene	EMT	Notch1-HEY1 pathway ↑ →drive self-renewal and EMT→increase proliferation, invasion and metastasis	MMPs	The SACC-LM cell line	^348^
NSCLC	Notch1, DLL4	Oncogene	Angiogenesis	ZLDI-8→suppress Notch1-HIF1α-VEGF signaling pathway→inhibit angiogenesis and vasculogenic mimicry	ZLDI-8, HIF1α, VEGF	The HUVECs, resistance A549/Taxol cells	^377^
CLL	DLL4, Notch1	Oncogene	Angiogenesis	DLL4→active Notch signaling→cell migration and angiogenesis↑	CXCR4	OP9-DLL1 and -DLL4 cells, Mouse OP9 BM stromal cells	^363^
Breast cancer	JAG1, DLL4, Notch1	Oncogene	Angiogenesis	linc-OIP5 ↓ → and JAG1 ↓ →disrupted DLL4/Notch/NRP1 signaling→suppress proliferation, migration, and tube formation	Linc-OIP5, YAP1, NRP1	MCF-10 cells, MDA-MB-231 cells, MCF-7 cells, HUVECs	^371^
Breast cancer	JAG1	Oncogene	Angiogenesis	JAG1 ↑ → MALAT1-miR-1405p-JAG1/VEGFA pathway ↑ →microenvironment angiogenesis↑	MALAT1, miR-140-5p, VEGFA	MCF-10A, T47D, MCF-7, MDA-MB-231 (231), MDA-MB-231 Bone (231B), HUVEC	^370^
Glioma	Notch1	Oncogene	Angiogenesis	DGC-secreted FMOD→activate integrin-dependent Notch1 signaling→promote angiogenesis	FMOD	GSCs MGG4, MGG6, and MGG8, DBT-Luc cells, ST1 endothelial cells, B.End3 cell line, The U251/U87/LN229 cell line	^376^
Glioma	DLL4, Notch1, NICD	Oncogene	Angiogenesis	Fibulin-3→active Notch signaling→DLL4 ↑ →promotes glioma angiogenesis	Fibulin-3	CNS1, GSCs GBM8 and GBM34, HBMECs	^364^
Melanoma	Notch1, N1ICD	Oncogene	Angiogenesis	Notch1 ↑ →CD133 ↑ →mitogen-activated protein kinase activation ↑ →melanoma growth, angiogenesis, and lung metastasis↑	CD133, p-p38	CD133 + , CD133- or unsorted B16F10-Luc cells	^375^
CRC	NICD	Oncogene	Stem-like properties	Lipids→recruit Numb→MDM2 degrade Numb→activate Notch signaling→promote stem-like cell features	Lipids, Numb, MDM2	HCT116, HT29	^387^
CRC	JAG2, Notch1, Notch2	Oncogene	Stem-like properties	tRF/miR-1280 ↑ →inhibit Notch signaling→suppress CSC phenotypes→suppress CRC growth and metastasis	tRF, miR-1280	HCT116, HCT15, HT29, Panc-1, 293 T cells	^388^
Liver cancer	Notch1	Oncogene	Stem-like properties	iNOS/NO→activate TACE/ADAM17→activate Notch1 signaling→aggressive cancer phenotype	iNOS/NO, TACE/ADAM17	HLE, MHCC-97H, PLC/PRF/5, HepG2	^393^
LUAD	Notch	Oncogene	Stem-like properties	HIF1ɑ-regulated miR-1275→ activate Wnt/beta-catenin and Notch signaling→enhance the stemness of LUAD→promote tumorigenicity, recurrence, and metastasis	HIF1ɑ, miR-1275	EBAS-2B, L78, H460, A549, GLC-82, SPC-A1, PC9, H1299, H1975, H2228, A549-luc, H1299-luc	^397^
Breast cancer	Notch1, Notch3, Notch4, Hey1	Oncogene	Stem-like properties	Syndecan-1→regulate IL-6/STAT3, Notch and EGFR signaling pathways→modulate CSC phenotype	Syndecan-1	SUM-149, SKBR3	^399^
Glioma	Notch1	Oncogene	Glioma stem cells stemness	Notch1 ↑ →lncRNA TUG1 ↑ →sponging miR-145 and recruiting polycomb→maintain stemness features	lncRNA TUG1, miR-145	1228-GSC, 222-GSC	^389^
Breast cancer	NICD, Hey2	Oncogene	Stem-like properties, chemoresistance	HIF2α ↑ →activate Wnt and Notch pathways→ promote stem phenotype conversion and induce resistance	HIF2α	MCF7, MDA-MB-231	^398^
PDAC	Notch3, Notch4	Oncogene	Stem-like properties	KLF10 ↓ →activate Notch signaling→promote stem cell phenotype and tumorigenesis	KLF10	BCRC 60284, BCRC 60139	^403^
GC	Notch1	Oncogene	Glycolysis, chemoresistance	Pyrimidine biosynthesis→augmente Notch signaling→critical glycolytic enzymes ↑ →enhance aerobic glycolysis→confer chemoresistance	c-Myc and PKM2	AGS, HGC-27	^434^
Lung cancer	Notch1	Oncogene	Glycolysis	Notch1/TAZ axis→inhibit cytotoxic T cell activity, promote aerobic glycolysis and immune escape	TAZ	A549, PC9	^416^
T-ALL	Notch1	Oncogene	Metabolic reprogramming	Notch1 signaling ↓ →inhibition of glutaminolysis→induce a metabolic shutdown	PTEN	(HEK) 293 T, HPB-ALL, DND41	^428^
OC	NICD	Tumor suppressor	Glycolysis	miR-101 and miR-26a ↑ → imposed glucose restriction on T cells→EZH2 ↓ →inhibit the Notch pathway→inhibit T cell survival→poor antitumor immunity	miR-101 and miR-26a, EZH2	HEK293T	^419^
CRC	Notch1	Oncogene	Tumor microenvironment	Notch1 ↑ →promote TGF-β-mediated neutrophil infiltration recruitment→drive metastasis	TGF-β	*villin*Cre^ER^; *Kras*^G12D/+^; *Trp53*^fl/fl^ *Rosa*26^N1icd/+^ organoids; villinCreER; *Apc*^fl/fl^; *Kras*^G12D/^+; *Trp53*^fl/fl^ *TgfbrI*^fl/fl^ organoids	^442^
HCC	HEY1	Oncogene	Hypoxia	Hypoxia→HIF-1 ↑ → HEY1 ↑ → PINK1 ↓ →reduced oxidative stress, increased HCC growth	HIF-1, PINK1	HeLa, PLC/PRF/5, HepG2, Hep3B	^461^
Lung cancer	Notch1	Tumor suppressor	Tumor microenvironment	Apoptotic lung cancer cells→trigger Notch1 signaling→WISP-1 production ↑ →preventing the migration and invasion	WISP-1	344SQ, A549, HCT116	^444^
Glioma	Notch1	Oncogene	Tumor immune microenvironment	NKAP ↑ →Notch1 ↑ →active Notch1-dependent immune-suppressive tumor microenvironment→promote glioma growth and invasion	NKAP, SDF-1, M-CSF	U251, U87, Gl261	^447^
Glioma	DLL1	Oncogene	Hypoxia	hypoxia→Notch signaling ↓ →HES1 and Hey1 ↓ →promote neuroendocrine differentiation	NSE, β3-tubulin	LNCaP, PC-3, Du145	^458^
CRC	Notch2, RBPJ	Oncogene	Chemoresistance	miR195-5p→Notch2 and RBPJ ↓ →reduced CRC cell sternness and chemoresistance	miR195-5p	SW480, SW620, HT-29, HCT-160	^486^
PC	Notch2	Oncogene	Chemoresistance	Midkine–Notch2 interaction→activated Notch signaling→increase chemoresistance	Midkine	PaCa 5061, 5072, and 5156; L3.6pl (19), BxPC-3, PANC-1 cells	^472^
PC	N1ICD	Oncogene	Chemoresistance	LINC00261 → N1DARP→inhibit USP10-Notch1 oncogenic signaling →inhibit chemoresistance	LINC00261, N1DARP, USP10	Aspc1, Bxpc3, Capan1, MiaPaCa2, Panc1, Sw1990, and Patu8988; HPNE; MDA-MB-231 and MCF-10A; A549 and BEAS-2B; HCT116 and NCM460	^476^
PC	Notch1	Oncogene	Chemoresistance	GAS41 → H2A.Z.2→Notch1 ↑ →GEM resistance	GAS41, H2A.Z.2	PANC-1, BXPC3, Capan-1, MiaPaCa-2, SW1990	^475^
LUAD	Notch1	Oncogene	Chemoresistance	Notch1/AP-1/miR-451/MDR-1 signaling axis→influence chemoresistance of LUAD	AP-1, miR-451, MDR-1	SPC-A1 and H1299	^480^
Breast cancer	Notch1	Oncogene	Chemoresistance	Exosomal miR-378a3p and miR-378d → EZH2/STAT3 signaling→activate WNT and Notch stemness pathways→induce drug resistance	miR-378a3p, miR-378d, EZH2, STAT3	CAL51, MDA-MB231, MCF-7	^483^
TNBC	Notch1	Oncogene	Chemoresistance	Notch1→MVP→activate AKT pathway→promote EMT→promote chemoresistance	MVP	MDA-MB-231, BT549, MCF-7, T47D, SKBR-3, HCC1937, ZR751	^484^
Skin tumor	Notch1	Tumor suppressor	N/A	Notch1 ↓ →Gli2 ↑ →basal-cell carcinoma-like tumors ↑ ; Notch1 ↓ →beta-catenin ↑ →skin carcinogenesis	Gli2, beta-catenin	Primary keratinocytes	^496^
Skin tumor	Notch1	Tumor suppressor	Tumor microenvironment	Notch1 ↓ →epidermal differentiation/barrier formation defects→skin carcinogenesis	N/A	N/A	^498^
Multifocal epithelial tumor	CSL/RBP-Jκ	Tumor suppressor	N/A	CSL/RBP-Jκ ↓ →c-Jun and c-Fos ↑ →promote tumor cell proliferation	c-Jun, c-Fos	Dermal fibroblasts	^499^
SCC	Notch1	Tumor suppressor	N/A	Notch1 ↑ →ROCK1/2 and MRCK α kinases ↓ →suppress SCC	p53, ROCK1/2, MRCK α kinases	SCC011, SCC012, SCC022, SCC028	^500^
SCLC	Notch	Tumor suppressor	N/A	Notch ↑ →hASH1 ↓ →cell cycle arrest	hASH1, p21, p27kip1, ERK1, ERK2, raf/MEK/MAPK	DMS53, NCI-H209, Low passage 293, NCI-H209/ΔRaf-1:ER cells	^502^
Forebrain tumor subtypes	Notch1, Notch2, RBPJ	Tumor suppressor	N/A	Notch1/Notch2/RBPJ ↓ →accelerate glioma	p53, Trp53	Platinum-E cells	^503^
Cervical cancer	Notch1	Tumor suppressor	N/A	Notch1 ↑ →SST, SSTR1 and SSTR2 ↑ →inhibit cervical cancer	SST, SSTR1 and SSTR2	HeLa, HeLa-GFP, HeLa-ICN1	^257^
K-ras–induced PDAC	Notch1	Tumor suppressor	N/A	Notch1 ↓ →increase tumor incidence and progression	K-ras	Primary pancreatic ductal cells	^504^
B-ALL	HES1	Tumor suppressor	N/A	HES1 ↑ → PARP1 ↑ →cell apoptosis	PARP1, bHLH	JM1, Nalm6, 697, SupT1, Molt4, HEK-293	^505^

*ESCC* esophageal squamous cell carcinoma, *HCC* hepatocellular carcinoma, *SCLC* small cell lung cancer, *HNSCC* head and neck squamous cell carcinoma, *ACC* adenoid cystic carcinoma, *NSCLC* non-small cell lung cancer, *CLL* chronic lymphocytic leukemia, *CRC* colorectal cancer, *LUAD* lung adenocarcinoma, *PDAC* pancreatic ductal adenocarcinoma, *GC* gastric cancer, *T-ALL* T cell acute lymphoblastic leukemia, *B-ALL* B cell acute lymphoblastic leukemia, *OC* ovarian cancer, *PC* pancreatic cancer, *TNBC* triple-negative breast cancer, *EMT* epithelial-mesenchymal transition, *TGF-β* transforming growth factor beta, *Zeb1* zinc finger E-box-binding homeobox 1, *SCC* squamous cell carcinoma, *Tspan5* tetraspanin-5, *MCAM* melanoma cell adhesion molecule, *RBPJ* recombining binding protein suppressor of hairless, *HEY1* hairy and enhancer of split 1, *HIF1α* hypoxia-inducible factor 1-alpha, VEGF vascular endothelial growth factor, *DLL4* delta-like ligand 4, *JAG1* Jagged-1, *linc-OIP5* long intergenic non-protein coding RNA OIP5-AS1, *MALAT1* metastasis-associated lung adenocarcinoma transcript 1, *miR* microRNA, *FMOD* fibromodulin, *DGC* discoidin domain-containing receptor 1, *CD133* cluster of differentiation 133, *MDM2* mouse double minute 2, *tRF* transfer RNA-derived fragment, *CSC* cancer stem cell, *iNOS* inducible nitric oxide synthase, *NO* nitric oxide, *TACE* tumor necrosis factor-alpha converting enzyme, *ADAM17* a disintegrin and metalloprotease 17, *IL-6* interleukin 6, *STAT3* signal transducer and activator of transcription 3, *EGFR* epidermal growth factor receptor, *lncRNA* TUG1 long non-coding RNA taurine upregulated gene 1, *KLF10* Krüppel-like factor 10, *USP24* ubiquitin-specific protease 24, *PLK1* polo-like kinase 1, *GSI* gamma-secretase inhibitor, *AKT* protein kinase B, *mTOR* mammalian target of rapamycin, *EZH2* enhancer of Zeste homolog 2, *PINK1* PTEN-induced putative kinase 1, *WISP-1* Wnt-induced signaling protein 1, *NKAP* NF-kappa-B-activating protein, *HES1* hairy and enhancer of split 1, *Hey1* Hes-related family bHLH transcription factor with YRPW motif 1, *RBPJ* recombining binding protein suppressor of hairless, *N1ICD* Notch1 intracellular domain, *GAS41* growth arrest-specific 41, *H2A.Z.2* histone H2A.Z.2, *GEM* gemcitabine, *MVP* major vault protein, *SST* somatostatin, *SSTR* somatostatin receptor, *PARP1* poly ADP-ribose polymerase1, *bHLH* basic-helix-loop-helix

### Notch signaling pathway in EMT

**Fig. 3:**
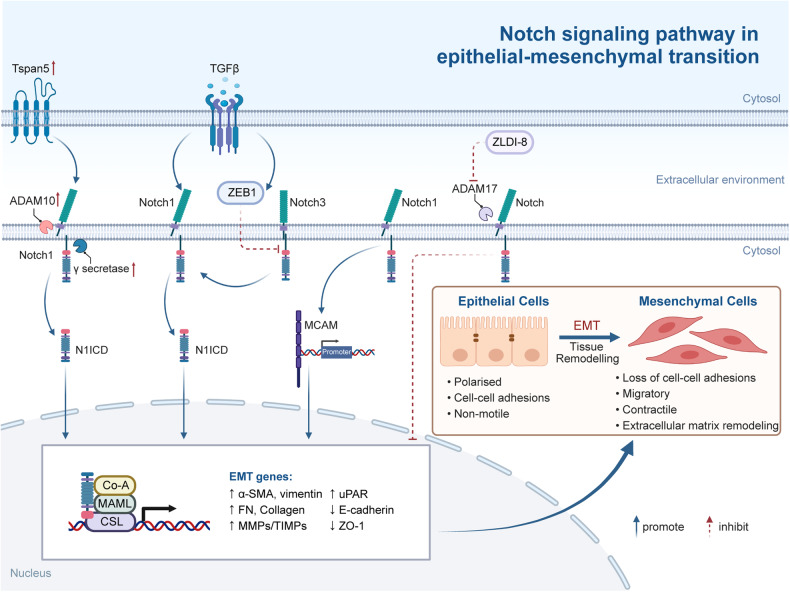
Notch signaling pathway in epithelial-mesenchymal transition (EMT). EMT is a complex process wherein epithelial cells undergo a transition, losing their inherent characteristics and adopting a mesenchymal phenotype. Notch signaling plays a crucial role in regulating EMT, representing a significant mechanism for tumor cells to acquire malignant properties. (Figure created using BioRender.com). ADAM a disintegrin and metalloprotease, N1ICD Notch1 intracellular domain, ZEB1 zinc finger E-box-binding homeobox 1, MCAM melanoma cell adhesion molecule, Co-A coactivator, MAML mastermind-like, CSL CBF1/suppressor of hairless/Lag1

EMT, originally described by Elizabeth Hays in the 1980s, denotes the intricate process wherein epithelial cells undergo a transformation, losing their characteristic features and adopting mesenchymal phenotypes.^338^ EMT is a fundamental occurrence in events such as embryogenesis and tissue repair.^339,340^ Over the years, it has been observed that EMT is reactivated during tumor progression, emerging as a pivotal mechanism for cancer cells to acquire malignant properties.^341,342^ Various signaling pathways participate in the regulation of EMT, including the Notch signaling pathway (Fig. 3).

Numerous studies have indicated that the activation of Notch1 promotes EMT in HCC, contributing to the acquisition of stem-like characteristics, as well as facilitating migration, invasion, and chemoresistance.^343,344^ Mechanistically, Xie and colleagues have noted that tetraspanin5 activates Notch signaling by enhancing the γ-secretase-catalyzed cleavage of the Notch1 receptor.^345^ This activation further promotes EMT and rearrangement of the actin cytoskeleton, ultimately fostering the metastasis of HCC. In squamous cell carcinoma (SCC), emerging evidence suggests that Notch1 functions as an EMT-promoting factor driven by TGF-β, while Notch3-mediated signaling restricts terminal differentiation.^346^ Another study has demonstrated that the Notch4-HEY1 pathway is specifically up-regulated in HNSCC, inducing proliferation, cisplatin resistance, and promoting EMT.^347^ Similarly, Xie et al. have shown that the Notch1-HEY1 pathway is specifically up-regulated in salivary adenoid cystic carcinoma (ACC), driving cell self-renewal and EMT.^348^ These findings hold significant potential to broaden our comprehension of the role of the Notch pathway in tumor EMT and may guide the development of new strategies to reverse EMT by targeting the Notch signaling pathway.

Primary drug resistance is commonly observed in cancer cells exhibiting mesenchymal differentiation.^349,350^ EMT is recognized as a contributor to chemotherapy resistance in various tumors, including NSCLC, breast cancer, and glioma.^351,352^ Consequently, effective inhibition of the Notch signaling pathway emerges as a promising strategy to overcome chemoresistance. CBF1, also known as RBPJ, has been identified as a participant in the EMT-like phenotype of glioma cells.^353^ Maciaczyk et al. demonstrated that inhibiting CBF1 can impede EMT activators, such as zinc finger E-box-binding homeobox 1, resulting in decreased cell invasiveness and chemoresistance in EMT-like glioblastoma cells. ZLDI-8, a novel inhibitor of ADAM17, has been reported to inhibit the Notch pathway and reverse the EMT process, thereby inhibiting migration and invasion in chemotherapy-resistant NSCLC.^354^ Notch1 induces EMT and chemoresistance in TNBC cells by directly activating the MCAM promoter. Down-regulation of Notch1 significantly inhibits MCAM expression, thereby reversing EMT and cisplatin chemotherapy resistance in TNBC cells. These studies collectively provide molecular evidence highlighting the impact sof Notch signaling-mediated EMT on tumor chemoresistance. Consequently, Notch inhibitors may prove to be effective anti-EMT therapies, offering a potential avenue to prevent chemoresistance in tumor cells.

### Notch signaling pathway in tumor angiogenesis

**Fig. 4:**
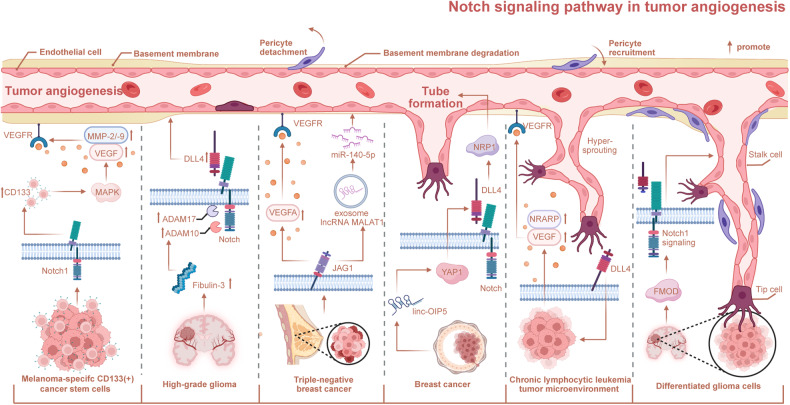
Notch signaling pathway in tumor angiogenesis. Abnormal angiogenesis, a distinctive feature of the tumor microenvironment, provides essential nutrients for tumor growth and facilitates the entry of malignant cells into circulation, leading to distant metastases. The Notch signaling cascade plays a crucial role in mediating tumor angiogenesis. (Figure created using BioRender.com). VEGFR vascular endothelial growth factor receptor, VEGF vascular endothelial growth factor, MAPK mitogen-activated protein kinase, DLL4 delta-like ligand 4, ADAM a disintegrin and metalloprotease, VEGFA vascular endothelial growth factor A, JAG1 Jagged1, NRP1 neuropilin 1, NRARP Notch-regulated ankyrin repeat protein, FMOD fibromodulin

The Notch signaling pathway in tumor angiogenesis is a significant aspect of the multi-stage process involved in the formation of new blood vessels from the original ones.^355^ This process is crucial for embryonic development, normal tissue growth, bone formation, and wound healing. Abnormal angiogenesis, a distinctive feature of the TME, provides essential nutrients for tumor growth and creates an opportunity for malignant cells to enter the circulation, forming distant metastases.^356,357^ Vascular endothelial growth factor (VEGF) is considered the central signaling mediator for angiogenesis.^358,359^ Moreover, the Notch signaling cascade has been demonstrated to play a crucial role in regulating tumor angiogenesis (Fig. 4).^360^ DLL4, a Notch ligand, modulates angiogenesis by controlling endothelial cell activation, vascular development, and maturation.^361,362^ Recent research by Mónica et al. indicates that DLL4 expressed in the TME can induce Notch signaling activation in Notch1-mutated CLL cells.^363^ Additionally, DLL4 triggers the expression of Notch-regulated ankyrin repeat protein and VEGF, leading to increased angiogenesis. Nandhu et al. discovered that Fibulin-3, a protein secreted by glioma cells, acts as a paracrine activator of Notch signaling, motivating angiogenesis in high-grade glioma.^364^ Mechanistically, Fibulin-3 enhances the expression of DLL4 in an ADAM10/17-dependent manner, thereby activating DLL4-Notch signaling.

JAG1, as a classic ligand of the Notch pathway, has been identified as playing a role in angiogenesis. However, accumulating evidence suggests that JAG1 and DLL4 influence different downstream signaling pathways, resulting in distinct vascular phenotypes.^365,366^ Generally, DLL4 inhibits endothelial cell sprouting by activating Notch signaling, leading to a sparse network of large-caliber vessels.^367^ In contrast, JAG1 mediates signal transduction in both tumor cells and endothelial cells, promoting vascular sprouting and higher vascular density.^368,369^ Liu et al. have preliminary evidence confirming JAG1’s pro-angiogenic effect in TNBC, possibly participating in angiogenesis through the enhancement of the MALAT1-miR-1405p-JAG1/VEGFA pathway.^370^ This suggests a potential synergistic effect between JAG1 and VEGFA in promoting angiogenesis. Another study has reported that JAG1 may mediate the long intergenic non-coding RNA linc-OIP5 to regulate the DLL4/Notch/NRP1 signaling pathway in human umbilical vein endothelial cells, affecting angiogenesis in the breast cancer microenvironment.^371^ However, further experiments are needed to explore how JAG1 interacts with Notch ligands such as DLL4 to regulate tumor angiogenesis.

Activated Notch1 signaling is frequently observed in endothelial cells of various human cancers, and this is positively correlated with worsened prognosis.^372^ Continuous activation of Notch1 alters the morphology and function of endothelial cells, promoting the migration of tumor cells across the vascular wall. Additionally, Notch1 signaling participates in the angiogenesis phenotype.^373,374^ Kumar et al. reported that Notch1 drives the expression of CD133, activates MAPK, and regulates the expression of MMP-2/-9 and VEGF in melanoma-specific CD133^+^ CSCs, leading to melanoma angiogenesis.^375^ Sengupta et al. revealed that differentiated glioma cells secrete the proteoglycan fibromodulin to promote glioma angiogenesis by activating Notch1 signaling.^376^ In drug-resistant NSCLC, the inactivation of the Notch1-HIF1α-VEGF pathway by ZLDI-8 suppresses angiogenesis and vasculogenic mimicry.^377^ Collectively, these findings contribute to a comprehensive understanding of the mechanism of the Notch pathway in mediating tumor angiogenesis and may enrich the therapeutic targets for tumors.

### Notch signaling pathway in CSLC properties

**Fig. 5:**
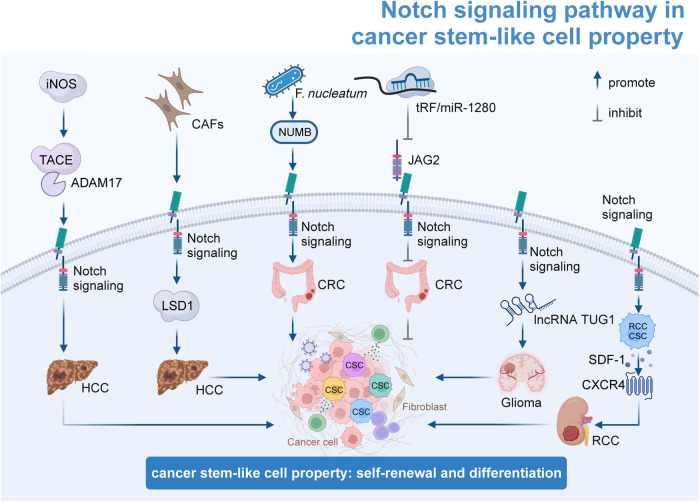
Notch signaling pathway in cancer stem-like cell (CSLC) properties. CSLC, a subset of tumor cells with notable self-renewal potential and multidirectional differentiation ability, are regulated by the abnormal activation of the Notch signaling pathway. (Figure created using BioRender.com). iNOS inducible nitric oxide synthase, TACE TNF-alpha converting enzyme, ADAM a disintegrin and metalloprotease, HCC hepatocellular carcinoma, CAFs cancer-associated fibroblasts, LSD1 lysine-specific histone demethylase 1A, CRC colorectal cancer, JAG2 Jagged2, SDF-1 stromal cell-derived factor-1, CXCR4 CXC chemokine receptor 4, RCC renal cell carcinoma, CSC cancer stem cell

CSCs, a subgroup of tumor cells with notable self-renewal potential and multidirectional differentiation ability, are increasingly recognized in various solid tumors.^378–380^ Their presence is considered a driver of malignancy initiation, metastasis, and chemotherapy resistance. Recent evidence suggests that non-CSCs can acquire stem-like properties in certain processes, such as EMT, abnormal activation pathways, expression of specific stem cell biomarkers, and immune escape.^381–383^ Abnormal activation of key signaling pathways controlling stem cell self-renewal, including the Notch signaling pathway, is deemed a crucial factor in regulating CSLC properties (Fig. 5).^384,385^ For instance, Xiao et al. reported that in RCC, activated Notch signaling can maintain the stemness of CSCs and promote their chemotaxis through the SDF-1/CXCR4 axis.^386^ This study provides new insights into how RCC CSCs maintain stemness through the Notch pathway. Liu et al. found that *Fusobacterium nucleatum* infection promotes the degradation of Numb mediated by lipid droplets, resulting in activated Notch signaling and the acquisition of stem-like properties in CRC cells.^387^ Targeting the Notch ligand JAG2, tRF/miR-1280 inactivates Notch signaling, suppressing the CRC stem-like phenotype and inhibiting tumor formation and metastasis.^388^ Katsushima et al. revealed the role of Notch signaling in maintaining the stemness of GSCs.^389^ Specifically, activated Notch1 in GSCs induces the expression of the long non-coding RNA TUG1, influencing the stemness of GSCs. In HCC, CSCs are implicated in treatment resistance and poor survival outcomes.^390,391^ Liu et al. demonstrated that Notch3 is essential for liver CSC self-renewal and tumor proliferation.^392^ CAFs maintain the stability of lysine-specific histone demethylase 1 A (LSD1) by inducing LSD1 deacetylation through Notch3 activation, accelerating the self-renewal of liver CSCs. Additionally, highly expressed inducible nitric oxide synthase activates Notch1 through the TACE/ADAM17 pathway, promoting the CSC phenotype and enhancing HCC aggressiveness.^393^ These groundbreaking findings illuminate the role of the Notch signaling pathway in coordinating the self-renewal of liver CSCs, with potential implications for improving treatment strategies and limiting recurrence.

CSLC properties rely on a complex interplay of multiple signaling pathways that form an interacting network.^394,395^ Studies have demonstrated that Notch signaling can synergistically interact with other biological processes, such as the WNT and EGFR pathways, to regulate CSLC phenotypes.^396^ For instance, Jiang et al. reported that HIF1α mediates the overexpression of miR-1275, activating both Wnt/β-catenin and Notch signaling pathways, thereby enhancing the stemness of LUAD cells.^397^ In a hypoxic TME, Yan et al. found that overexpressed HIF2α induces stem-like phenotypic transformation through the activation of Wnt and Notch pathways, increasing the resistance of breast cancer cells to PTX.^398^ Syndecan-1, identified as a novel molecular marker in inflammatory breast cancer, was shown to modulate CSLCphenotypes through the IL-6/STAT3, Notch, and EGFR signaling pathways.^399^ In ACC, a population of CD133^+^ cells with neural stem cell properties was identified, and Notch1 and SOX10 were found to drive the proliferation and radiation-resistance of CD133^+^ CSCs.^400^ Lin et al. observed significant upregulation of Notch4 protein in melanoma CSLCs (MCSLCs), where Notch4^+^ MCSLCs promoted metastasis and invasion by initiating the EMT process.^401^ Krüppel-like factor 10 (KLF10), a zinc finger-containing transcription factor, was revealed to inhibit Notch3 and Notch4 transcription by binding to the promoter of E74-like ETS transcription factor 3.^402^ KLF10 deficiency led to the development of a PDAC stem-like phenotype and tumorigenesis by promoting the Notch signaling pathway.^403^ Up-regulation of KLF10 or inhibition of Notch signaling at the gene level or pharmacologically reduced the stem-like phenotype and tumor growth in PDAC. To fully exploit the therapeutic potential of targeted Notch signaling in malignant tumors, further research is required to explore the intricate crosstalk between Notch signaling and core components of other pathways. This exploration aims to identify potential balances for regulating stem-like phenotypes in cancer cells.

### Notch signaling pathway in cancer metabolic reprogramming

**Fig. 6:**
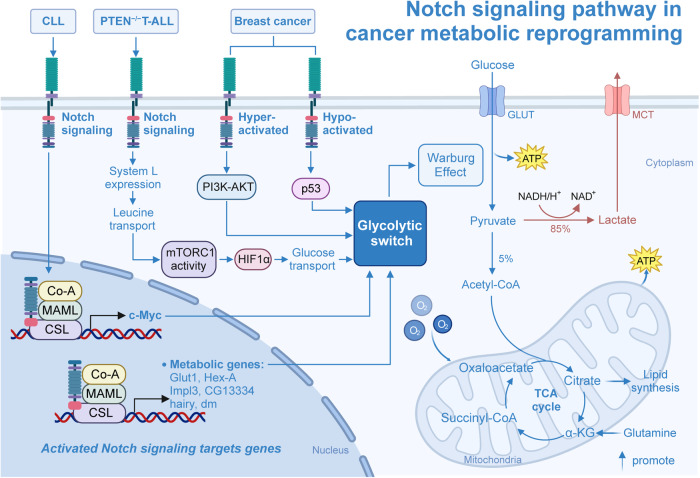
Notch signaling pathway in cancer metabolic reprogramming. Notch signaling plays a crucial role in the metabolic reprogramming of cancer cells, particularly during the glycolytic switch. The Notch pathway is active during this transition in cancer cells, with several genes directly regulated as transcriptional targets. This regulation mediates the shift in cellular metabolism towards the Warburg effect. (Figure created using BioRender.com). CLL chronic lymphocytic leukemia, PTEN phosphatase and tensin homolog, T-ALL T cell acute lymphoblastic leukemia, PI3K/AKT phosphoinositide 3-kinase/protein kinase B, HIF1α hypoxia-inducible factor 1α, Co-A coactivator, MAML mastermind-like, CSL CBF1/suppressor of hairless/Lag1, GLUT glucose transporters, MCT monocarboxylate transporter, ATP adenosine triphosphate, NADH nicotinamide adenine dinucleotide hydride, NAD nicotinamide adenine dinucleotide, TCA tricarboxylic acid, α-KG alpha**-**ketoglutarate

Metabolic reprogramming is a prominent feature of cancer, encompassing alterations in glucose, lipid, and amino acid metabolism. The Warburg effect, first described by Warburg in the 1920s, highlighted that tumor cells preferentially undergo glycolysis even in the presence of oxygen.^404–406^ Although less efficient in ATP production, aerobic glycolysis supports the rapid proliferation and survival of malignant cells.^407,408^ The Notch signaling pathway plays a pivotal role in the metabolic reprogramming of cancer cells (Fig. 6). Previous studies have demonstrated that Notch signaling becomes active during the glycolytic switch in cells.^409^ Several genes involved in controlling glycolysis and the tricarboxylic acid cycle, such as Glut1, Hex-A, Ecdysone-inducible gene L3, Impl3, and hairy, are direct transcriptional targets of the Notch pathway, mediating the transition of cellular metabolism toward the Warburg effect. Jitschin et al. found that stromal cells facilitate glycolytic switching in CLL cells through the Notch-c-Myc signaling pathway, leading to an increase in aerobic glycolysis.^410,411^ Another study showed that hyper-activated and hypo-activated Notch signaling induces glycolytic switching in breast tumor cells through different mechanisms.^412^ Specifically, hyper-activated Notch signaling promotes glycolysis in a PI3K/AKT-dependent manner, while hypo-activated Notch signaling weakens mitochondrial respiratory chain activity and enhances glycolysis via the p53-dependent pathway. In T-ALL cells, activated Notch signaling drives high System L amino acid transporter activity, promoting leucine transport and uptake, which, in turn, enhances glucose transport via the mTORC1/HIF1α pathway.^413^ Additionally, Notch coordinates c-Myc and mTORC1-controlled metabolic reprogramming, increasing glutamine transport. Sellers et al.^414^ found that the two main subtypes of NSCLC, SCCs and adenocarcinomas, exhibit distinct metabolic reprogramming. Upregulated Notch activity is associated with altered metabolic phenotypes in lung SCC. Mouse lung tumors driven by Notch and MYC reproduce the SCC-specific metabolic reprogramming characteristics. These studies collectively suggest that Notch signaling is a crucial regulator of energy metabolism in malignant cells and is required to maintain metabolic flexibility.

Lactic acid, a byproduct of aerobic glycolysis, contributes to the increased acidity of the TME. In the presence of a low pH environment, anti-tumor effector cells, such as T cells, are prone to functional loss and apoptosis. Growing evidence suggests that the acidity of the TME plays a crucial role in regulating tumor immunity, orchestrating local and systemic immunosuppression.^415^ Consequently, oncogene-induced metabolic reprogramming may be linked to immune escape. For instance, Xie and colleagues uncovered that Notch1 promotes the expression of glycolytic genes through interaction with histone acetyltransferases p300 and pCAF.^416^ Moreover, Notch1 signaling and PDZ-binding motif form a positive feedback loop that elevates extracellular lactate levels, inhibiting the activity of cytotoxic T cells and ultimately contributing to the malignant behavior of lung cancer. The shift from oxidative phosphorylation to glycolysis is known to be crucial for the activation and effector function of memory T cells.^417^ Given the limited nutrient availability in tumors, it is reasonable to hypothesize that T cell glycolytic metabolism undergoes changes in the TME.^418^ Zhao et al. found that Notch signaling is implicated in effector T cell dysfunction mediated by the methyltransferase EZH2 in OC.^419^ Mechanistically, OC cells restrict the aerobic glycolysis of T cells by maintaining high expression of miR-101 and miR-26a. These specific miRNAs inhibit EZH2 expression, target Notch repressors, and promote Notch activation, thereby attenuating T cell-mediated anti-tumor immunity. Collectively, these studies unveil the connection between Notch signaling, cancer metabolic reprogramming, and immune escape. Targeting Notch signaling holds the potential to enhance the efficacy of immunotherapy by inhibiting aerobic glycolysis.

The heightened Warburg effect not only fosters the proliferation and metastasis of tumor cells but also bestows various tumor characteristics that contribute to drug resistance.^420,421^ These characteristics include increased drug efflux, mutations in drug targets, inactivation of drug metabolism, and enhanced DNA damage repair, among others. Recent studies have shed light on specific mechanisms linking the Warburg effect to drug resistance in different cancers. For example, a study revealed that the ACYP1/HSP90/MYC/LDHA axis promotes the Warburg effect, driving sorafenib resistance in HCC.^422^ Another investigation found that Aldo-keto reductase family 1 B10 enhances the Warburg effect, marked by excessive lactic acid production, leading to acquired chemotherapy resistance in lung cancer brain metastasis to pemetrexed.^423^ Additionally, inhibition of HIF1α-mediated aerobic glycolysis and mitochondrial dysfunction can restore the sensitivity of tamoxifen in the treatment of breast cancer.^424^ Notch signaling has also been implicated in mediating chemotherapy resistance through metabolic reprogramming.^425^ Notch1-induced glutaminolysis is identified as a key carbon source for T-ALL and a determinant of anti-Notch1 therapeutic response in vivo.^426,427^ Inhibition of Notch1 in T-ALL results in metabolic impairment, significant inhibition of glutaminolysis, and induction of autophagy to provide essential metabolites to leukemia cells.^428^ The mutational loss of the tumor suppressor PTEN, a negative regulator of the PI3K/AKT signaling pathway, can promote glycolysis and induce drug resistance against anti-Notch1 treatment in T-ALL.^428,429^ This emphasizes the critical role of Notch1 signaling in controlling leukemia cell metabolism and glutaminolysis as a therapeutic target in Notch1-induced T-ALL. Furthermore, cancer cells have a high demand for pyrimidine nucleotides to support accelerated DNA and RNA synthesis.^430,431^ Thus, they heavily rely on the de novo synthesis approach of pyrimidine.^432,433^ He et al. found that de novo pyrimidine synthesis enhances the expression of key glycolytic enzymes and promotes aerobic glycolysis by activating Notch signaling and c-Myc gene transcription, conferring GC cells with chemotherapy resistance.^434^ This underscores the pivotal role of pyrimidine de novo synthesis in aerobic glycolysis and identifies it as a metabolic vulnerability that can be targeted to overcome chemotherapy resistance in GC.

### Notch signaling pathway in TME

**Fig. 7:**
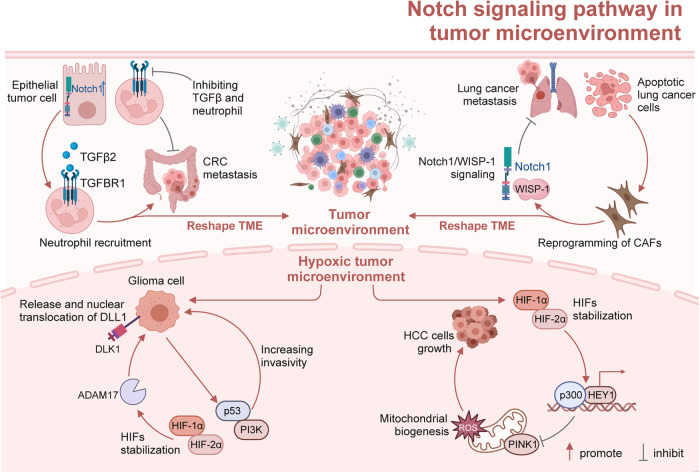
Notch signaling pathway in tumor microenvironment (TME). The Notch signaling pathway actively participates in the components of the TME, regulating TME through both paracrine and autocrine signals. (Figure created using BioRender.com). TGF-β2 transforming growth factor-beta2, TGF- βR1 transforming growth factor-beta receptor 1, CRC colorectal cancer, DLL1 delta-like ligand 1, ADAM a disintegrin and metalloprotease, HIF hypoxia-inducible factor, PI3K phosphoinositide 3**-**kinase, WISP Wnt-induced signaling protein 1, CAFs cancer-associated fibroblasts, HCC hepatocellular carcinoma, PINK1 PTEN-induced putative kinase 1, ROS reactive oxygen species

The malignant characteristics of cancer rely on the bidirectional interaction between cancer cells and their environment, giving rise to a well-organized complex ecosystem known as the TME.^435^ The TME consists of tumor cells, blood vessels, immune cells, stromal cells, and extracellular matrix, forming a dynamic and intricate network.^436^ The crosstalk between cancer cells and their environment involves various signaling pathways, including NF-κB,^437^ TGF-β,^438^ cGAS-STING signaling,^439,440^ and the Notch pathway.^441^ Notably, the Notch signaling pathway plays a crucial role in shaping the components of the TME, regulating it through paracrine or autocrine signals (Fig. 7). In a recent study by Jackstadt et al., the activation of Notch1 signaling in mouse intestinal epithelium was found to reshape the TME of CRC, showing a close association with poor prognosis.^442^ Moreover, activated Notch1 signaling was shown to promote the metastasis of Kras^G12D^-driven serrated cancer through TGF-β-dependent neutrophil recruitment. Tumor growth and metastasis driven by locally activated neutrophils was also observed in the lung microenvironment, which is governed by enhanced Notch signaling.^443^ Significantly, targeting Notch-driven neutrophil recruitment might be an effective strategy in preventing cancer metastasis. CAFs represent crucial stromal cells in the TME, capable of reshaping the extracellular matrix environment and promoting tumor progression and metastasis through paracrine communication. Kim et al.^444^ demonstrated that apoptotic lung cancer cells, induced by ultraviolet irradiation, can reprogram CAFs, enhance the secretion of Wnt-induced signaling protein 1 by activating Notch1 signaling, and subsequently inhibit the migration and invasion of both cancer cells and CAFs. This study underscores the context-dependent role of activated Notch signaling in either promoting or inhibiting carcinogenesis within the TME.

The immune components within tumors collectively form the tumor immune microenvironment (TIME), which includes innate immune cells, adaptive immune cells, and cytokines.^445,446^ The TIME has been shown to play a crucial role in the initiation and progression of tumors. Gu et al. reported that the overexpression of NF-kappa B activating protein directly binds to the Notch1 promoter and transactivates it, contributing to glioma growth by promoting the Notch1-dependent immunosuppressive TME.^447^ Advancements in high-throughput and high-dimensional technologies, such as spatial transcriptome and proteome analyses, have allowed researchers to describe the spatial architecture of the TIME and explore its functions in tumor biology.^448^ Single-cell RNA sequencing has revealed the remodeling of myeloid cells and lymphocytes in the TIME during tumor dormancy.^449^ Specifically, the JAG1/Notch signaling pathway was found to regulate immune homeostasis during dormant minimal residual disease.^449^ In another study, the single-cell landscapes of the human liver, from development to disease, were examined. The research found that VEGF/Notch signaling pathways mediate an immunosuppressive onco-fetal TME in HCC.^450^ Further investigation revealed a common immunosuppressive microenvironment between fetal liver and HCC, particularly involving VEGF/Notch signaling in the re-emergence of fetal-associated endothelial cells (i.e., PLVAP/VEGFR2) and fetal-like (i.e., FOLR2) tumor-associated macrophages.^451^ This concept of an immunosuppressive onco-fetal TME mediated by VEGF/Notch signaling provides a potential new target for immunotherapy of HCC.

The hypoxic microenvironment is a prominent and common feature in many solid tumors, including PC,^452^ HCC,^453^ breast cancer,^454^ and melanoma.^455^ Hypoxia plays a role in mediating the malignant biological behavior of cancer cells and can impact the therapeutic outcomes of tumors through complex mechanisms.^456,457^ In glioma cells, Grassi et al.^458^ revealed that hypoxia induces the release of intracellular fragments of DLL1, a Notch ligand, which is dependent on ADAM17 and HIF1α/HIF2α. Interestingly, hypoxic glioma cells exhibit unexpected nuclear translocation of DLL1, leading to altered activation of the p53 and PI3K pathways and increased aggressiveness of gliomas. Hypoxia often leads to inefficient electron transfer in the mitochondrial electron transport chain, resulting in the accumulation of reactive oxygen species (ROS). The downstream signaling triggered by HIF activation is a key molecular mechanism for cells to adapt to hypoxia.^459,460^ Chiu et al. found that the HIF1 and Notch signaling pathways interact to control mitochondrial biogenesis in cancer cells and maintain redox balance.^461^ Specifically, HIF1 directly binds to the hypoxia response element of HEY1 in the Notch signaling pathway, activating the transcription of HEY1 in HCC. HEY1, in turn, inhibits the expression of PTEN-induced putative kinase 1 (PINK1), reducing the production of mitochondrial ROS and promoting the growth of HCC. Therefore, the HIF1/HEY1/PINK1 pathway confers a survival advantage on HCC in the hypoxic microenvironment.

### Notch signaling pathway in chemoresistance

**Fig. 8:**
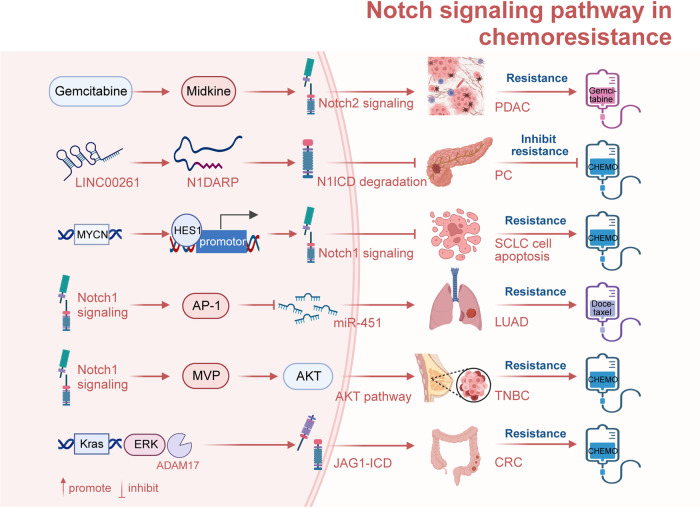
Notch signaling pathway in chemoresistance. The aberrant expression and overactivation of Notch pathway components play crucial roles in contributing to chemoresistance. (Figure created with BioRender.com). PDAC pancreatic ductal adenocarcinoma, N1DARP Notch1 degradation-associated regulatory polypeptide, N1ICD Notch1 intracellular domain, CHEMO chemotherapy, SCLC small cell lung cancer, AP-1 activator protein-1, LUAD lung adenocarcinoma, MVP major vault protein, AKT protein kinase B, TNBC triple-negative breast cancer, ADAM a disintegrin and metalloprotease, JAG1-ICD Jagged1 intracellular domain, CRC colorectal cancer

Chemotherapy is the traditional treatment for all types of cancer.^462^ Despite the development of numerous novel chemotherapy strategies, response rates to treatment for many advanced tumors remain low due to the emergence of intrinsic or acquired chemoresistance.^463,464^ Various mechanisms can confer chemoresistance in cancer,^465,466^ including decreased drug activity, elevated efflux of anticancer agents, alterations in drug targets, changes in DNA repair mechanisms, and evasion of drug-induced apoptosis. Emerging evidence suggests that acquired chemoresistance may also involve complex mechanisms, such as the development of EMT-like phenotypes in cancer cells, metabolic reprogramming, stem cell characterization, and alterations in molecular pathways.^467,468^ Identifying specific signaling pathways that are abnormally activated in chemoresistance is crucial for adjusting therapeutic regimens. Therefore, exploring the molecular processes behind chemoresistance is essential for improving tumor treatment outcomes. Aberrant expression of components in the Notch pathway is known to play a crucial role in contributing to chemoresistance (Fig. 8).^469,470^

Previous research has shown that excessive activation of the DLL4/Notch pathway in PDAC causes defective angiogenesis within tumors, resulting in low efficiency of chemotherapeutic drug delivery in vivo and enhanced multi-drug chemoresistance.^471^ Another study revealed that GEM, a first-line chemotherapy agent for PDAC, induces Midkine expression in a dose-dependent manner.^472^ Furthermore, Midkine interacts with Notch2 to activate Notch signaling, driving PDAC resistance to GEM.^472^ Similar to PDAC, chemotherapy resistance is a major challenge for PC. Cumulative data suggest that activation of the Notch signaling pathway contributes to PC resistance to GEM. Inhibition of Notch signaling is reported to enhance the chemosensitivity of PC to GEM by activating the intrinsic apoptotic pathway.^473^ Glioma amplified sequence 41 (GAS41) is reported to be a novel regulator of Notch signaling by controlling H2A.Z deposition.^474^ Han et al. found that GAS41 binds to H2A.Z.2 to activate Notch1 signaling and its downstream mediators, driving PC stemness and GEM resistance.^475^ Given the function of widely activated Notch signaling in chemotherapy resistance of PC cells, Notch signaling is expected to become a potential therapeutic target for PC. Zhai et al. identified a new microprotein, Notch1 degradation-associated regulatory polypeptide (N1DARP), encoded by LINC00261.^476^ N1DARP promotes N1ICD degradation by destroying USP10-N1ICD interactions, thereby suppressing chemoresistance in Notch1-overactivated PC. These findings provide a promising alternative strategy for PC and may have widespread application in a variety of malignancies.

Chemoresistance in lung cancer is a multifactorial process involving the dysfunction of oncogenes and tumor suppressors in various signaling pathways, such as Notch.^477,478^ A recent study revealed that MYCN binds to the HES1 promoter, activating the Notch pathway.^479^ This activation inhibits drug-induced apoptosis and enhances chemotherapy resistance in SCLC. Similarly, Li et al. reported that chemo-resistant NSCLC cells acquire a more invasive phenotype through EMT and dysregulated Notch pathways. Furthermore, molecular evidence demonstrated that terfenadine reverses epirubicin sensitization via EMT and the Notch pathway. Huang and colleagues uncovered that Notch1 negatively regulates miR-451 through activator protein-1, influencing the proliferation and apoptosis of LUAD and conferring chemoresistance to docetaxel (DTX).^480^ Inhibition of Notch1 sensitized LUAD to DTX, suggesting that combining DTX with a GSI could be a novel strategy for treating DTX-resistant LUAD. Notably, beyond PC and lung cancer, Notch signaling also mediates the sensitivity of breast cancer and CRC cells to chemotherapy agents.^481,482^ Yang et al. demonstrated that cytotoxic drugs used in neoadjuvant therapy for breast cancer can stimulate the secretion of exosomes by cancer cells, promoting chemotherapy resistance through the activation of WNT/β-catenin and Notch stem cell pathways in vivo.^483^ Additionally, Xiao et al. revealed that Notch1 positively regulates the transcription of major vault protein, activating the AKT pathway, promoting the EMT process, and participating in chemotherapy resistance in TNBC cells.^484^ In CRC with a Kras mutation, JAG1 has been shown to trigger intrinsic reverse signal transduction through its nuclear-targeted JAG1-ICD, maintaining the progression and chemoresistance of CRC.^485^ Another study demonstrated that miR-195-5p inhibits CRC cell stemness and 5-fluorouracil resistance by inhibiting Notch2 and RBPJ.^486^ In summary, inhibiting Notch pathways holds promise for restoring chemotherapy efficacy in CRC.^487^

### Notch signaling pathway in tumor suppression

**Fig. 9:**
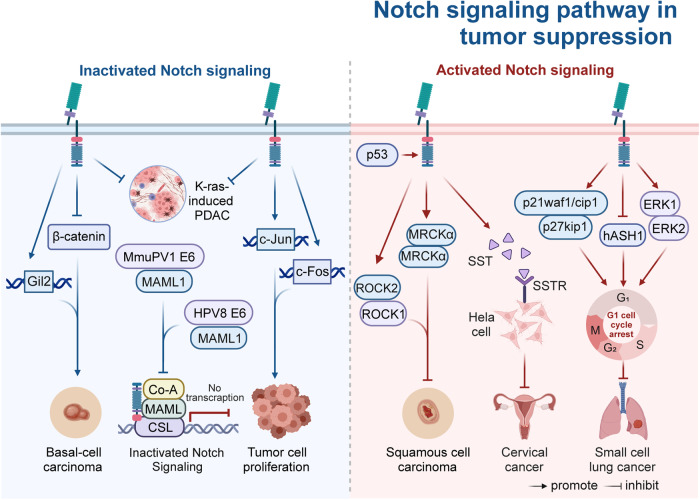
Notch signaling pathway in tumor suppression. Earlier studies provided evidence supporting Notch-mediated tumor suppression in various malignancies, including SCC, cervical cancer, and SCLC. (Figure created using BioRender.com). PDAC pancreatic ductal adenocarcinoma, MmuPV1 Mus musculus papillomavirus 1, HPV8 human papillomavirus, Co-A coactivator, MAML mastermind-like, CSL CBF1/suppressor of hairless/Lag1, hASH1 human achaete-scute homolog-1, SST somatostatin, SSTR somatostatin receptor, SCC squamous cell carcinoma, SCLC small cell lung cancer

Research on the role of Notch signaling pathway during tumorigenesis primarily centers on its function as an oncogene. Increasing evidence suggests that Notch signaling also acts as a tumor suppressor in various malignancies, including SCC, hematological malignancies, cervical cancer, and forebrain glioma (Fig. 9).^488–491^ The oncogenic or tumor-suppressive role of the Notch signaling pathway is believed to be greatly dependent on the environment.^492,493^ As a crucial form of intercellular communication, Notch signaling regulates the differentiation of keratinocytes and maintains skin homeostasis.^494,495^ Nicolas and colleagues found that Notch1 functions as a tumor suppressor gene in mammalian skin.^496^ Deficiency of Notch1 in mouse skin and primary keratinocytes results in elevated Gli2 expression and improper activation of beta-catenin signaling, ultimately leading to skin carcinogenesis. Another study found that E6 proteins of the cancer associated human papillomavirus (HPV) 8 and Mus musculus papillomavirus 1(MmuPV1) can bind to the Notch co-activator MAML to inhibit Notch signaling, which is associated with delayed differentiation and sustained keratinocyte proliferation.^497^ Additionally, Demehri et al. demonstrated that the tumor-promoting effect of Notch1 deletion in epidermal keratinocytes involves the impaired skin-barrier integrity and wound-like skin microenvironment.^498^ In human skin samples, suppressed Notch/CSL signaling was observed in stromal fields surrounding multifocal premalignant actinic keratosis lesions, while gene expression of c-Jun and c-Fos was upregulated.^499^ Moreover, Notch1 is a p53 target gene and participates in the inhibition of human aggressive SCC by negatively regulating ROCK1/2 and MRCKα kinases.^500^ Wu et al. found that PTC124 (Ataluren) could help HNSCC cells re-express functional Notch1 to substitute the nonsense mutation Notch1, thus preventing the proliferation of HNSCC cells.^501^ Taken together, these findings provide evidence that Notch functions as tumor suppressor in relation to SCC, and SCC may be a cancer subtype that could benefit from specific activation of the Notch receptor.

In addition to SCC, earlier studies have offered evidence supporting Notch-mediated tumor suppression in several solid malignancies. SCLC exhibits typical neuroendocrine characteristics, dependent on the involvement of the basic-helix-loop-helix transcription factor known as human achaete-scute homolog-1 (hASH1). Previous research has indicated that the activated Notch signaling pathway suppresses the expression of hASH1 in SCLC cells, leading to cell cycle arrest and growth inhibition linked to the p21waf/cip1 and ras signaling pathway.^502^ Giachino et al. discovered that Notch signaling acts as a tumor suppressor in forebrain tumor subtypes.^503^ Their findings indicate that Notch signaling can collaborate with p53 to inhibit cell proliferation and tumor growth in grades II-III astrocytoma, proneural glioblastoma, and supratentorial primitive neuroectodermal tumor. In a stable Notch1-activated cervical cancer HeLa cell line established by Laura and colleagues, activation of Notch1 led to apoptosis, cell cycle arrest, and tumor suppression.^257^ Mechanistically, Notch1-mediated tumor suppression in cervical cancer may be partly achieved by up-regulating somatostatin (SST) signaling. A prior study also revealed an unforeseen tumor suppressor role for Notch1 in a K-ras-induced PDAC murine model, where K-ras is activated and Notch1 is deleted.^504^ In this model, the absence of Notch1 results in increased tumor occurrence and advancement, suggesting that Notch1 may act as a tumor suppressor in K-ras-induced PDAC. With respect to B-ALL, Notch target HES1 triggers the activation of poly ADP-ribose polymerase1 (PARP1), leading to B-ALL cell apoptosis in a cell type-specific manner.^505^ These findings suggest that Notch signaling might regulate the fate of tumor cells in a context-dependent way through various intricate mechanisms. Importantly, the functions of Notch signaling as a tumor suppressor could contribute to the advancement of Notch agonist-based cancer treatments.

## Ongoing therapeutic strategies targeting Notch signaling in human malignancies

**Fig. 10:**
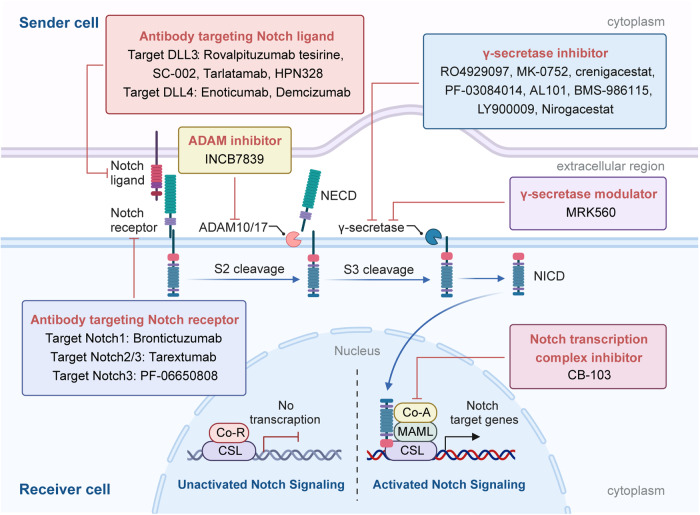
Therapeutic strategies targeting Notch signaling in human malignancies. Various pharmacological agents aimed at the Notch pathway have been developed, including γ-secretase inhibitors (GSIs), ADAM inhibitors, antibodies against Notch receptors or ligands, inhibitors targeting the Notch transcription complex, and γ-secretase modulators (GSMs). (Figure created with BioRender.com). DLL3 delta-like ligand 3, DLL4 delta-like ligand 4, ADAM a disintegrin and metalloprotease, NECD Notch extracellular domain, NICD Notch intracellular domain, Co-A coactivator, MAML mastermind-like, CSL CBF1/suppressor of hairless/Lag1

Efforts to develop therapeutic strategies targeting Notch signaling continues unabated, with numerous drug studies currently progressing through preclinical or clinical trials for various human malignancies. Researchers have devised a range of Notch-targeted therapies for each stage of the Notch signaling cascade, as illustrated in Fig. 10. In this context, we provide a summary of specific inhibitors and blocking antibodies currently undergoing clinical trials for Notch signaling, encompassing GSIs, ADAM inhibitors, antibodies targeting Notch receptors or ligands, Notch transcription complex inhibitors, and γ-secretase modulators (GSMs) (Table 3).

**Table 3:** Therapeutic strategies targeting Notch signaling in clinical trials against human malignancies

Type	Agents	Cancer	Identifier	Enrollment (N)	Phase	Status	Country	First posted	Ref.
γ-Secretase inhibitor	RO4929097	Metastatic CRC	NCT01116687	37	Phase II	Completed	United States	2010	^506^
	RO4929097	Previously treated metastatic PDAC	NCT01232829	37	Phase II	Completed	United States	2010	^507^
	RO4929097	Metastatic melanoma	NCT01120275	36	Phase II	Terminated	United States	2010	^508^
	RO4929097	Recurrent platinum-resistant EOC	NCT01175343	45	Phase II	Completed	United States, Canada	2010	^509^
	RO4929097	Recurrent/progressive Glioblastoma	NCT00980343	47	Phase II	Completed	United States	2010	^510^
	RO4929097	Advanced solid tumors	NCT01198184	18	Phase Ib	Completed	Canada	2010	^511^
	RO4929097	Glioma	NCT01119599	21	Phase 0/I	Completed	United States	2010	^512^
	RO4929097	Recurrent malignant glioma	NCT01189240	13	Phase I	Terminated	United States	2010	^513^
	RO4929097	Metastatic breast cancer	NCT01149356	15	Phase Ib	Terminated	United States	2010	N/A
	RO4929097	Advanced sarcoma	NCT01154452	78	Phase Ib/II	Completed	United States	2010	^514^
	MK-0752	PDAC	NCT01098344	44	Phase I	Completed	United Kingdom	2010	^518^
	MK-0752	Advanced solid tumors	NCT01243762	47	Phase I	Terminated	USA, Canada, Israel	2010	^520^
	MK-0752	Advanced solid tumors	NCT01295632	28	Phase I	Completed	United States; France; etc.	2011	^519^
	Crenigacestat (LY3039478)	Advanced or metastatic ACC	NCT01695005	22	Phase I	Completed	United States, Denmark, etc.	2012	^521^
	Crenigacestat	Advanced or metastatic cancer	NCT01695005	28	Phase I	Completed	United States, Denmark, etc.	2012	^522^
	Crenigacestat	Advanced or metastatic solid tumors	NCT02784795	63	Phase Ib	Completed	United States, Denmark, etc.	2016	^523^
	Crenigacestat	Advanced solid tumors	NCT02836600	16	Phase I	Completed	Japan	2016	^524^
	Crenigacestat	Advanced or metastatic solid tumors	NCT02784795	31	Phase Ib	Completed	United States, Denmark, etc.	2016	^525^
	Crenigacestat	T-ALL and T-LLy	NCT02518113	36	Phase I	Completed	United States, France, etc.	2015	^527^
	PF-03084014	T-ALL and T-LLy	NCT00878189	8	Phase I	Completed	United States, Italy	2009	^528^
	PF-03084014	Advanced solid malignancies	NCT00878189	64	Phase I	Completed	United States, Italy	2009	^526^
	AL101	TNBC	NCT04461600	67	Phase II	Active, not recruiting	United States, Belgium, etc.	2020	N/A
	AL101	ACC	NCT04973683	14	Phase Ib	Recruiting	Texas	2021	N/A
Selective γ-secretase inhibitor	BMS-986115	Advanced solid tumors	NCT01986218	36	Phase I	Terminated	United States, Australia, etc.	2013	^529^
	LY900009	Advanced cancer	NCT01158404	35	Phase I	Completed	United States	2010	^530^
	Nirogacestat (PF-03084014)	Desmoid tumor/aggressive fibromatosis	NCT03785964	142	Phase 3	Active, not recruiting	United States, Belgium, etc.	2019	^531^
ADAM inhibitor	INCB7839	Metastatic HER2^+^ breast cancer	NCT01254136	20	Phase I/II	Terminated	United States	2010	N/A
	INCB7839	HER2-positive metastatic breast cancer	NCT00864175	68	Phase I/II	Terminated	India	2007	N/A
	INCB7839	Recurrent/ progressive high-grade gliomas	NCT04295759	13	Phase I	Active, not recruiting	United States	2020	N/A
mAb targeting Notch1	Brontictuzumab (OMP-52M51)	Solid tumors	NCT01778439	48	Phase I	Completed	United States	2013	^535^
Cross-reactive antibody targeting Notch2/3	Tarextumab (OMP-59R5)	Solid tumors	NCT01277146	42	Phase I	Completed	United States	2010	^536^
	Tarextumab	Untreated metastatic PC	NCT01647828	177	Phase II	Completed	United States	2012	^537^
ADC targeting Notch3	PF-06650808	Breast cancer and other advanced solid tumors	NCT02129205	40	Phase I	Terminated	United States	2014	^538^
ADC targeting DLL3	Rovalpituzumab tesirine (Rova-T)	Recurrent SCLC	NCT01901653	82	Phase I	Completed	United States	2013	^539^
	Rovalpituzumab tesirine	Advanced, recurrent SCLC	NCT03086239	29	Phase I	Completed	Japan	2017	^540^
	Rovalpituzumab tesirine	Extensive-stage SCLC	NCT02819999	28	Phase I	Terminated	United States	2016	^541^
	Rovalpituzumab tesirine	DLL3-expressing, relapsed/refractory SCLC	NCT02674568	342	Phase II	Completed	United States, France, etc.	2016	^542^
	Rovalpituzumab tesirine	Previously-treated extensive-stage SCLC	NCT03026166	42	Phase I-II	Terminated	United States, France, etc.	2017	^543^
	Rovalpituzumab tesirine	Extensive-stage SCLC	NCT03033511	748	Phase III	Terminated	United States, Australia, etc.	2017	^544^
	Rovalpituzumab tesirine	DLL3-high SCLC	NCT03061812	444	Phase III	Completed	United States, Australia, etc.	2017	^545^
	Rovalpituzumab tesirine	DLL3-expressing advanced solid tumors	NCT02709889	200	Phase I/II	Terminated	United States	2016	^546^
	SC-002	Relapsed or refractory SCLC and LCNEC	NCT02500914	35	Phase I	Terminated	United States	2015	^547^
BiTE targeting DLL3	Tarlatamab (AM757)	SCLC	NCT03319940	392	Phase I	Recruiting	United States, Australia, etc.	2017	N/A
TriTAC targeting DLL3	HPN328	Advanced cancers	NCT04471727	162	Phase I/II	Recruiting	United States	2020	N/A
mAb targeting DLL4	Enoticumab (REGN421)	Advanced solid tumors	NCT00871559	83	Phase I	Completed	United States	2009	^548^
	Demcizumab (OMP-21M18)	Previously-treated solid tumors	NCT00744562	42	Phase I	Completed	United States	2008	^549^
	Demcizumab (OMP-21M18)	Metastatic non-squamous NSCLC	NCT01189968	46	Phase Ib	Completed	Australia, New Zealand, etc.	2010	^550^
	Demcizumab (OMP-21M18)	Platinum-resistant EOC	NCT01952249	20	Phase Ib	Terminated	United States	2013	^551^
Notch transcription complex inhibitor	CB-103	Advanced or metastatic solid tumors and hematological malignancies	NCT03422679	79	Phase I/II	Terminated	United States, France, etc.	2017	^553^

*CRC* colorectal cancer, *PDAC* pancreatic ductal adenocarcinoma, *EOC* epithelial ovarian cancer, *ACC* adenoid cystic carcinoma, *T-ALL* T cell acute lymphoblastic leukemia, *T-LLy* T cell lymphoblastic lymphoma, *TNBC* triple-negative breast cancer, *PC* pancreatic cancer, *SCLC* small cell lung cancer, *LCNEC* large cell neuroendocrine carcinoma, *ADAM* a disintegrin and metalloprotease, *mAb* monoclonal antibody, *ADC* antibody-drug conjugate, *BiTE* bispecific T cell engager, *TriTAC* tri-specific T cell activating construct, *DLL3* delta-like ligand 3

### γ-Secretase inhibitors

Given their therapeutic potential in inhibiting Notch signaling in specific cancers, GSIs are actively being explored as cancer therapeutic drugs. Over the past decade, the antitumor activity of at least eight GSIs has undergone investigation in early-stage clinical trials across various tumor types. RO4929097, a Notch signaling GSI, has been a focus of clinical studies since 2010, assessing its efficacy in patients with advanced tumors. Phase II trials revealed limited clinical activity for RO4929097 as a standalone treatment in advanced tumors, including metastatic CRC,^506^ previously treated metastatic PDAC,^507^ metastatic melanoma,^508^ recurrent platinum-resistant EOC,^509^ and recurrent/progressive glioblastoma.^510^ As a result, RO4929097 was deemed insufficient for further single-drug study, with common mild toxicity including fatigue, nausea, and anemia. However, when combined with immunosuppressants or monoclonal antibodies, RO4929097 demonstrated good tolerance in the treatment of advanced solid tumors.^511–513^ Notably, its combination with endocrine therapy for endocrine-resistant Erα-positive breast cancer warrants further investigation. Unfortunately, a Phase Ib/II trial investigating the effects of RO4929097 and the hedgehog inhibitor vismodegib in advanced sarcoma did not observe any objective responses in patients.^514^ MK0752, used as a single drug in Phase I clinical trials for advanced solid tumors and children with refractory CNS malignancies, exhibited good tolerance.^515–517^ The most common drug-related adverse events included diarrhea, nausea, vomiting, and fatigue. In PDAC, the combination of MK-0752 with GEM showed a satisfactory evaluation of tumor response in 44 patients receiving the recommended Phase II dose.^518^ However, combining MK-0752 with the mTOR inhibitor ridaforolimus or insulin growth factor 1 receptor pathway inhibitors demonstrated clinical activity accompanied by drug-related adverse events such as diarrhea and rash.^519,520^ Crenigacestat (LY3039478) underwent Phase I clinical trials, alone or in combination with different anticancer drugs, in advanced or metastatic solid tumors and T-ALL and T cell lymphoblastic lymphoma (T-Lly).^521–527^ However, the clinical efficacy was disappointing, and crenigacestat treatment frequently resulted in dose-limiting toxicities such as fatigue, diarrhea, nausea, and vomiting. Oral GSI PF-03084014 demonstrated antitumor activity in advanced solid malignancies and T-ALL/T-Lly, supporting further evaluation of its clinical application.^528^ The primary drug-related toxicities of PF-03084014 include diarrhea, nausea, fatigue, and hypophosphatemia, typically ranging from mild to moderate in severity. Ongoing clinical trials are assessing the efficacy and safety of AL101 monotherapy in patients with Notch-activated recurrent or metastatic TNBC, as well as the potential benefits of AL101 before surgery for treating Notch-activated ACC. Results from these trials are awaited. Selective GSIs BMS-986115 and LY900009, explored in Phase I clinical trials, have shown safety and good tolerance for advanced tumors, exhibiting sustained targeting and biological activity in inhibiting Notch signaling.^529,530^ Furthermore, a Phase III clinical trial of the oral selective GSI nirogacestat (PF-03084014) demonstrated significant benefits in PFS and objective reflection of progressing desmoid tumors.^531^

### ADAM inhibitor

The metalloproteinases ADAM10 and ADAM17 play a crucial role in cleaving Notch receptors, initiating downstream signaling that contributes to maintaining the invasive characteristics of tumors.^532,533^ Consequently, targeted inhibition of ADAM10 or ADAM17 represents a crucial approach to halting the progression of malignant tumors. INCB7839, an inhibitor of ADAM10 and ADAM17 proteases, has undergone assessment in Phase I–II clinical trials for previously treated solid tumors and breast cancer. However, Phase I clinical trials revealed that INCB7839 monotherapy displayed restrictive toxicity, including deep vein thrombosis, along with adverse events such as fatigue and nausea. An ongoing multi-center Phase I clinical trial is investigating INCB7839 targeting microenvironmental neuroligin-3 in the treatment of recurrent or progressive high-grade gliomas, with results yet to be reported. More recently, Nayanendu et al. developed a human anti-ADAM10 monoclonal antibody (mAb) named 1H5.^534^ Preclinical studies have shown that 1H5, when combined with the chemotherapeutic drug Irinotecan, effectively inhibits tumor growth in colon cancer mice without causing obvious toxic effects. Consequently, researchers hypothesize that mAb-mediated ADAM10 inhibition is a promising method to specifically prevent drug resistance and metastasis in CRC.

### Antibodies targeting Notch receptors or ligands

While GSIs have exhibited robust therapeutic potential in clinical trials, their significant limitation lies in the inhibition of all Notch receptors. Consequently, highly specific mAbs targeting individual Notch receptors or ligands have been developed to address this challenge. Brontictuzumab (OMP-52M51) is a mAb that specifically targets Notch1, inhibiting the activation of the Notch pathway. In a Phase I clinical trial involving 48 subjects with solid tumors, the investigation focused on determining the maximum tolerated dose (MTD) and preliminary efficacy of brontictuzumab.^535^ The results indicated that brontictuzumab was well tolerated at the MTD, with diarrhea identified as the main adverse reaction, attributed to the targeted effect of Notch1 inhibition. Tarextumab (OMP-59R5) is a novel cross-reactive antibody that binds to and selectively inhibits Notch2 and Notch3 signaling pathways. In the treatment of solid tumors, tarextumab demonstrated general tolerability with dose-limited diarrhea.^536^ However, when combined with nab-PTX and GEM, tarextumab did not improve the survival of untreated metastatic PC.^537^ Additionally, PFS in patients treated with tarextumab was statistically worse. PF-06650808 is a novel anti-Notch3 antibody-drug conjugate (ADC). In a Phase I clinical trial involving 40 patients with advanced breast cancer and other advanced solid tumors, PF-06650808 displayed early signs of manageable safety and anti-tumor activity.^538^ The most common adverse reactions in patients treated with PF-06650808 were gastrointestinal symptoms such as decreased appetite, nausea, and abdominal pain, as well as fatigue, alopecia, and pruritus. Rovalpituzumab tesirine (Rova-T) is an ADC targeting DLL3, expressed in over 80% of SCLC. While the Phase I clinical trial of Rova-T monotherapy for recurrent SCLC demonstrated encouraging anti-tumor activity and manageable safety, subsequent Phase II and Phase III trials indicated a lack of survival benefits in extensive-stage SCLC.^539–545^ Additionally, Rova-T was associated with toxicities such as serosal effusion, photosensitivity, and peripheral edema. In another Phase I/II clinical trial involving 200 patients with DLL3-expressing advanced solid tumors, Rova-T exhibited controllable toxicity at the recommended Phase II dose.^546^ Anti-tumor activity was observed in patients with neuroendocrine carcinomas/neuroendocrine tumors, melanoma, MTC, and glioblastoma. SC-002, another DLL3-directed ADC, showed systemic toxicity and limited efficacy in Phase I clinical trials for the treatment of advanced SCLC and large cell neuroendocrine carcinoma.^547^ Tarlatamab (AM757) is a half-life extended bispecific T cell engager (BiTE^®^) targeting DLL3. A Phase I study is currently evaluating the safety, tolerability, and pharmacokinetics of tarlatamab in patients with SCLC. HPN328, a tri-specific T cell activating construct (TriTAC^®^) targeting DLL3, is undergoing a Phase I/II trial to assess safety, tolerability, and pharmacokinetics, both as monotherapy and in combination with atezolizumab, in patients with advanced cancer associated with DLL3 expression. Enoticumab (REGN421) is a fully human IgG (1) mAb that binds to human DLL4, disrupting Notch-mediated signal transduction. In a Phase I trial, enoticumab was well tolerated in the treatment of advanced solid tumors, with observed treatment responses.^548^ Demcizumab (OMP-21M18) is an IgG2 humanized mAb targeting DLL4. Phase I clinical trials suggested that demcizumab is generally well tolerated and exhibits anti-tumor activity in previously treated solid tumors.^549^ Subsequent Phase IB clinical trials revealed that 50% of patients with metastatic non-squamous NSCLC had an objective tumor response after treatment with the truncated demcizumab regimen.^550^ In platinum-resistant EOC, demcizumab combined with PTX demonstrated controllable toxicity and activity in patients with severely pretreated platinum-resistant patients with OC.^551^

### Notch transcription complex inhibitors

Notch signaling initiates downstream cascades by guiding the formation of core transcriptional activation complexes. In addition to targeting the upstream components of the Notch signaling cascade through GSIs or antibodies that disrupt the interaction between Notch receptors and ligands, inhibiting transcriptional activation complexes offer an attractive approach to prevent Notch signal transduction.^552^ CB-103, the first small molecule drug developed to effectively inhibit the Notch transcription complex, underwent a Phase I/II clinical trial involving 79 adult patients with advanced or metastatic solid tumors and hematological malignancies.^553^ The trial demonstrated that CB-103 was well-tolerated, with 19% of patients experiencing grade 3–4 treatment-related adverse events, including elevated liver function, anemia, and visual changes, 6% of patients discontinued treatment due to toxicity. Recent studies have revealed that CB-103 has in vitro antitumor activity in a small subset of lymphoma cell lines from various lymphoma subtypes, with activity surpassing that achieved by GSIs.^554^ In preclinical models of endocrine-resistant and TNBC, CB-103, when combined with fulvestrant or PTX, exhibits synergistic effects, effectively inhibiting the formation of breast spheroids.^555^ Moellering et al. introduced a hydrocarbon-stapled peptide named SAHM1, which can prevent the assembly of active transcription complexes.^556^ In vivo and in vitro experiments have demonstrated that SAHM1 can treat T-ALL cells by globally suppressing Notch-activated genes. Another study identified a small molecule inhibitor, mastermind recruitment-1 (IMR-1), which targets the inhibition of the Notch transcription-activating complex.^557^ IMR-1 represents a potential new paradigm for Notch-based anticancer therapy.

### γ-Secretase modulators

GSMs have emerged as preferable drug candidates in response to the observed toxicity linked to non-selective GSIs in clinical trials. Unlike GSIs, GSMs do not inhibit gamma-secretase itself; instead, they are designed to regulate the catalytic activity of gamma-secretase, thereby influencing the function of Notch signaling.^558,559^ The initial objective in the quest for GSMs was to diminish the production of the 42-amino acid amyloid β peptide variant in the brains of patients with Alzheimer’s disease, without impeding the hydrolysis of Notch protein or causing the accumulation of carboxy-terminal fragments of the amyloid precursor protein.^560,561^ A preclinical study demonstrated that MRK560, a GSM targeting PSEN1, effectively reduced the processing of Notch1 mutants and induced cell cycle arrest without causing intestinal toxicity in T-ALL animal models.^562^ In certain scenarios, GSMs may present a potential alternative to GSIs, but further preclinical trials and clinical studies are still required to validate their efficacy and safety.

## Conclusions and perspectives

Since the initial discovery of the Notch protein family, our understanding of the Notch signaling pathway has deepened significantly. Despite the simple structure of the Notch signaling cascade, which involves only a few steps from ligand binding to initiating downstream target gene transcription, the biological functions of the Notch signaling pathway are complex and diverse in different systems. Overall, the effects of the Notch signaling heavily rely on the cellular environment and involve intricate crosstalk with other signaling pathways. The studies summarized in this review provide compelling evidence that Notch signaling pathway plays a considerable role in human malignancies. Given the complex oncogenic or tumor suppressive functions of the Notch signaling pathway in different malignancies, it is of great significance to focus on understanding the mechanisms through which the Notch signaling pathway regulates tumorigenesis and development.

Present research areas include the regulation of the Notch signaling pathway in tumors via biological processes like EMT, angiogenesis, and cancer metabolic reprogramming. However, it is vital to acknowledge that this investigation field is complicated, and related molecular mechanisms have not been extensively studied. In particular, the Notch signaling cascade is a crucial tumor suppressor in multiple cellular contexts and cancer types. Further studies are needed to delve into the precise mechanism of Notch-mediated tumor suppression, which will be beneficial for developing novel therapeutic strategies.

Currently, various inhibitors targeting γ-secretase, ADAM, and the Notch transcription complex, as well as antibodies targeting Notch receptors and ligands, have been proposed to control tumor progression. While early clinical trials have shown that therapies targeting the Notch signaling pathway exhibit some antitumor activity, the development of safe, effective, and tumor-specific Notch-targeted drugs for clinical use remains a significant challenge. It is important to note that tumors with Notch inactivating mutations, like HNSCC, are not appropriate for “anti-Notch” treatment approaches. Conversely, Notch receptor-specific antibody agonizts could be clinically valuable in tumors where Notch signaling acts as a tumor suppressor. Moreover, solely targeting the Notch signaling pathway may prove insufficient for effective cancer treatment. The combination of Notch-targeted drugs with immune checkpoint inhibitors, anti-angiogenic agents, or chemotherapy holds the promise of enhancing synergistic therapeutic effects. Continued research in this area is essential for unlocking the full potential of Notch-targeted therapies in cancer treatment.

To optimize drug development efforts based on Notch signaling, several strategies can be considered. First, combining Notch-targeted therapy with carrier-based nanomaterials may enhance drug delivery efficiency.^563^ Second, a deeper exploration of the role of Notch signaling in regulating the TME can inform the design of immune therapies centered on Notch signaling. For instance, packaging Notch-targeted drugs into oncolytic viruses and releasing them into the TME can inhibit the recruitment and activation of immune-suppressive cells.^564^ Moreover, comprehensive research on the complex interaction networks between Notch signaling and pathways such as Hedgehog and Wnt can provide more compelling evidence for rational combination therapies.^565^

It is noteworthy that the advancement of high-throughput sequencing technology and artificial intelligence holds the potential to elucidate the molecular mechanisms of the Notch signaling pathway in specific tumors. This could offer new perspectives on the pathogenesis and therapeutic targets of cancer. The exponential growth of our understanding of the Notch signaling pathway in tumor biology over the past two decades underscores the need to translate this fundamental science into clinical practice. The time is ripe for harnessing this knowledge to advance more effective and targeted cancer therapies.
